# Length-dependent motions of SARS-CoV-2 frameshifting RNA pseudoknot and alternative conformations suggest avenues for frameshifting suppression

**DOI:** 10.21203/rs.3.rs-1160075/v1

**Published:** 2022-01-04

**Authors:** Shuting Yan, Qiyao Zhu, Swati Jain, Tamar Schlick

**Affiliations:** ‡ Department of Chemistry, New York University, New York, NY 10003 U.S.A.; ¶ Courant Institute of Mathematical Sciences, New York University, New York, NY 10012 U.S.A.; § NYU-ECNU Center for Computational Chemistry, NYU Shanghai, Shanghai 200062, P.R. China

## Abstract

Conserved SARS-CoV-2 RNA regions of critical biological functions define excellent targets for anti-viral therapeutics against Covid-19 variants. One such region is the frameshifting element (FSE), responsible for correct translation of viral polyproteins. Here, we analyze molecular-dynamics motions of three FSE conformations, discovered by graph-theory analysis, and associated mutants designed by graph-based inverse folding: two distinct 3-stem H-type pseudoknots and a 3-way junction. We find that the prevalent H-type pseudoknot in literature adopts ring-like conformations, which in combination with 5′ end threading could promote ribosomal pausing. An inherent shape switch from “L” to linear that may help trigger the frameshifting is suppressed in our designed mutant. The alternative conformation trajectories suggest a stable intermediate structure with mixed stem interactions of all three conformations, pointing to a possible transition pathway during ribosomal translation. These observations provide new insights into anti-viral strategies and frameshifting mechanisms.

## Introduction

In less than two years, COVID-19 with its novel infectious agent SARS-CoV-2 has already caused more than 266 million infections and 5 million deaths worldwide. Although the development of multiple vaccines has provided hope for a post-pandemic world, new virus variants with higher infectivity and increased ability to evade the immune system require us to maintain vigilance. Thus, the identification of novel anti-viral therapeutic targets and development of drugs against them remains a priority.

The single stranded SARS-CoV-2 RNA genome of 29,891 nucleotides includes two overlapping, shifted open reading frames ORF1a and 1b, which encode for viral polyproteins that begin the viral protein production. To correctly translate both polypeptides, the virus utilizes programmed −1 ribosomal frameshifting (−1 PRF) to stall and backtrack the ribosome by one nucleotide to bypass the stop codon near the start site of ORF1b.

**Figure 1:**
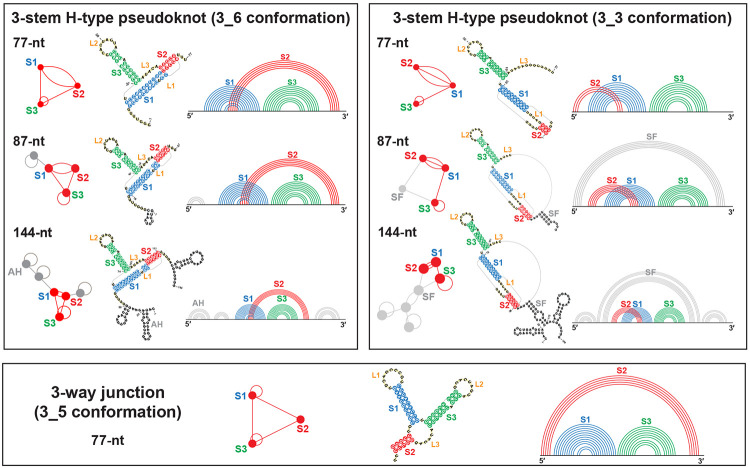
Secondary structures of the three FSE conformations at different lengths we study, along with their arc plots and corresponding dual graphs. For 77-nt, the three conformations 3_6 pseudoknot, 3_3 pseudoknot, and 3_5 junction have common Stems 1 (blue) and 3 (green), while different Stem 2 (red). The two pseudoknots are classified as H-type,^14^ where in 3_6 the loop region of Stem 1 binds with the external single-stranded 3′ end, and in 3_3 the Stem 1 loop binds with the 5′ end. For 87-nt, 10 upstream residues are added that include the 7-nt slippery site, and the 3_3 conformation contains an extra flanking stem SF (grey). For 144-nt, 37 upstream and 30 downstream residues are included, and extra stems (grey) are formed, including attenuator hairpin AH for 3_6 and SF for 3_3. Stems are represented as vertices in dual graphs, and loops as edges, with the central 3_6, 3_3, and 3_5 submotifs corresponding to the 77-nt FSE region highlighted in red, and the flanking vertices/edges corresponding to the extra stems/loops in grey.

First discovered in the *Rous sarcoma virus* in 1985,^1^ the – 1 PRF stalling of the ribosome is associated with a small (<100-nt) frameshifting element.^2^ SARS-CoV-2 similarly employs such a frameshifting element (FSE) located at the ORF1a/1b junction. This FSE consists of a 7-nt slippery site and a downstream 77-nt stimulatory region, which typically folds into an H-type pseudoknot (Fig. 1). The functional importance and high conservation of the FSE make it a promising candidate for anti-viral drugs and gene therapy; for example, in the latest Omicron variant, there are 31 new mutations in the spike gene region with respect to the previous variants, but no change in the FSE (Fig. S1).^3-6^ Whether frameshifting is orchestrated by the FSE acting as a “road blocker” or through more complex conformational switches remains unknown.^7-13^ Hence, exploring the secondary (2D) and tertiary (3D) structural dynamics of the FSE during translation is essential for both untangling the frameshifting mechanism and developing anti-viral strategies.

Unlike the stem-loop structure for HIV-1 FSE^15^ or the 2-stem pseudoknot for IBV FSE,^16^ the assumed structure for SARS-CoV-2 FSE is a 3-stem H-type pseudoknot, where the Stem 1 loop binds the 3′ end to form Stem 2, and Stem 3 lies between them (Fig. 1). This motif has been reported by chemical probing, Cryo-EM, NMR, crystallography,^3,17-23^ and molecular dynamics (MD).^24-26^ The Cryo-EM studies also suggest an “L” shape pseudoknot with coaxial stacking of Stems 1 and 2 which form the pseudoknot, and an extruding Stem 3 in the coaxial plane.^20,21^ In contrast, a recent crystallography study observes a vertical stacking of the 3 stems.^23^ Using our coarse-grained RNA-As-Graphs (RAG) representation as dual graphs,^27-30^ where double-stranded stems are vertices and single-stranded loops are edges, we assign this pseudoknot motif as dual graph 3_6 (Fig. 1).^3,25^ RAG, used to identify key RNA motifs, design novel RNA motifs from building blocks, and perform inverse folding to transform one RNA motif into another,^31-36^ was applied to explore and alter the FSE conformational landscape.^3,25^

Indeed, recent works revealed the complexity of the FSE landscape, with alternative conformations including different pseudoknots^3,20,37,38^ and unknotted structures^3,19,20,39-42^ (see^3^ for a detailed comparison). In particular, our prior modeling and SHAPE chemical reactivity experiments reveal an alternative 3-stem H-type pseudoknot where the Stem 1 loop binds with the 5′ end to form a different Stem 2 (3_3 dual graph), and a 3-way junction where the 5′ and 3′ ends pair (3_5 dual graph).^3^ The three conformations (3_6, 3_3, and 3_5) have common Stems 1 and 3 (though stem lengths vary) but competing Stem 2 (see Fig. 1). Moreover, our studies have emphasized the high length dependence of the FSE conformations: for short lengths such as 77-nt without the slippery site, the 3_6 pseudoknot is the dominant conformation, and the 3_5 junction is minor; for long lengths such as 87-nt and 144-nt, conformations containing the 3_6 pseudoknot become minor, while those containing the 3_3 pseudoknot become dominant.^3^ As in other positive-sense RNA viruses,^43-45^ structural transitions among these three (and other possible) conformations likely exist and play an important role in frameshifting.

Here we employ several computational 3D structure prediction programs and analyze microsecond MD trajectories of different FSE conformations at three lengths: 77, 87, and 144-nt. We term a particular conformation 3_6, 3_3, or 3_5 according to the central 77-nt FSE fold (Fig. 1). We consider all three conformations for the 77-nt FSE, and 3_6 and 3_3 conformations for 87 and 144-nt FSE. We also study our motif-strengthening mutants that stabilize each conformation over the others.

We identify structural features and motions that help suggest frameshifting mechanisms. For the 3_6 pseudoknot, the 5′ end threading through a ring hole formed by Stem 1 strand and junctions could add mechanical resistance to ribosomal unwinding and promote longer ribosomal pausing. The axial bending motion of the 3_6 pseudoknot we capture — from an “L” shape (observed by Cryo-EM^20,21^) to a more linear shape (observed by crystallography^23^) — can cause fluctuations in mRNA tension during translation, which might in turn trigger frameshifting. Importantly, our motif-strengthening mutant suppresses this motion and stabilizes the linear shape.

The large number of different motifs and RNA lengths modeled here for the first time allows us to piece observations and relate them to ribosomal translation. In shorter 3_3 systems, triplet hydrogen bonds that include Stem 2 interactions in all three conformations are present. Such a stable state suggests a potential transition among alternative FSE conformations as different sequence lengths are accessible to the ribosome. Namely, starting from longer sequences, where a flanking stem SF favors 3_3, a transition to 3_6 occurs when the ribosome occludes the slippery site to unwind SF and thus allow formation of alternative Stem 2.

These mechanistic findings hold specific implications for anti-viral strategies. Our work suggests targeting regions that participate in: 3_6 threading (3′ helix end of Stem 1), structural switch (Stem 2/3 junction), and pseudoknot stabilizing interactions (hydrogen-bonded triplets near Stem 2). Small molecules or gene editing mutations in these regions could hamper frameshifting.

## Results

### Overview

We model each FSE conformation using several 3D prediction programs (see Methods) and choose representative systems to discuss as follows: RNAComposer models for 3_6 pseudoknot, because they agree well with Cryo-EM experimental structures;^20,21^ iFoldRNA models for 3_3, because they maintain the 3_3 motif for all lengths; SimRNA models for 3_5, because they possess the elongated 3_5 structures seen in most systems. All systems can be found in the Supplementary Information with full descriptions.

**Figure 2:**
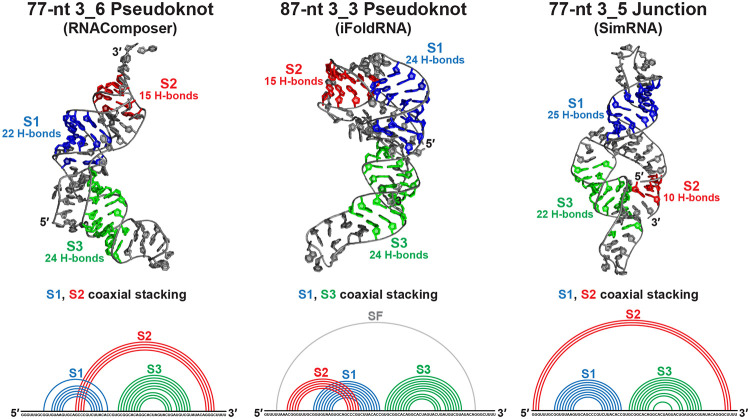
Representative structures for the three FSE conformations. The 77-nt 3_6 pseudoknot (largest MD cluster center structure from RNAComposer) has Stems 1 and 2 coaxially stacked. The 87-nt 3_3 pseudoknot (iFoldRNA) has Stems 1 and 3 coaxially stacked. The 77-nt 3_5 junction (SimRNA) has Stems 1 and 2 coaxially stacked. The 2D structures are extracted using 3DNA-DSSR,^46^ and the numbers of hydrogen bonds formed in the stems (averaged over the last 500 ns of the simulations) are calculated using Gromacs.^47^

Comparing the three representative structures (77-nt for 3_6 and 3_5, and 87-nt for 3_3) in Fig. 2, we note different helical arrangements and stem lengths. The 3_6 pseudoknot has Stems 1 and 2 coaxially stacked, while Stem 3 extrudes from the coaxial plane, forming an “L” shape. The 3_3 pseudoknot has Stems 1 and 3 stacked, and the pseudoknot is more compact. The 3_5 junction has Stems 1 and 2 stacked, but its Stem 2 is near Stem 3 instead of at the top. In all three structures, Stem 2 is much shorter than Stems 1 and 3, and 3_5 has the weakest Stem 2 consisting of mainly G-U wobble base pairs.

**Figure 3:**
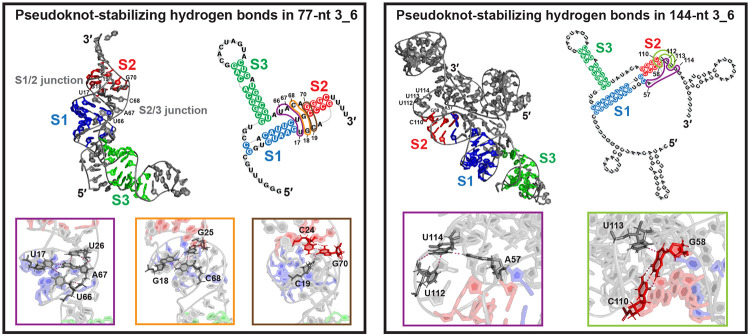
Pseudoknot stabilizing hydrogen bonds in our 3_6 systems. (Left) For 77-nt (RNAComposer), a base quadruplet and two triplets are formed at the Stem 1/2 and 2/3 junctions. (Right) For 144-nt (RNAComposer), two base triplets are formed at the 3′ helix end of Stem 2.

**Figure 4:**
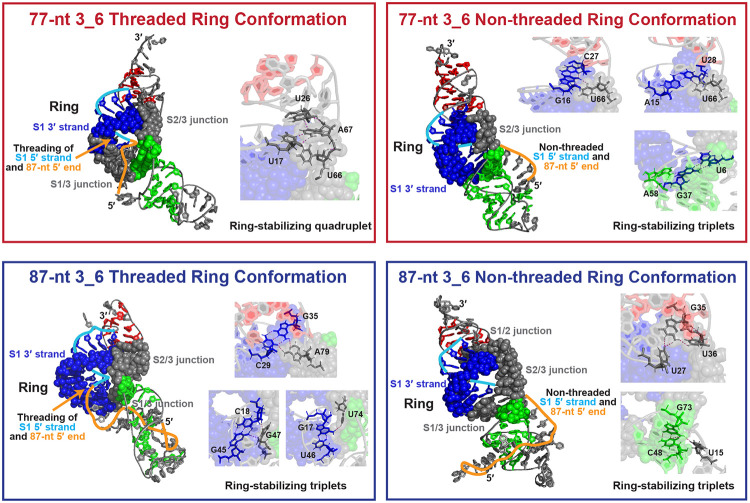
Threaded and non-threaded 3_6 pseudoknot ring conformations. (Top) The threaded 77-nt 3_6 structure (left, RNAComposer), with ring formed by the 3′ strand of Stem 1, and the Stem 1/3 and 2/3 junctions; the 5′ strand of Stem 1 and the 5′ end thread through the ring. A ring-stabilizing quadruplet formed at the ring top is enlarged. The non-threaded 77-nt 3_6 structure (right, iFoldRNA) has three ring-stabilizing triplets (two at top, one at bottom). (Bottom) Threaded 87-nt (left, RNAComposer) and non-threaded 87-nt (right, iFoldRNA).

**Figure 5:**
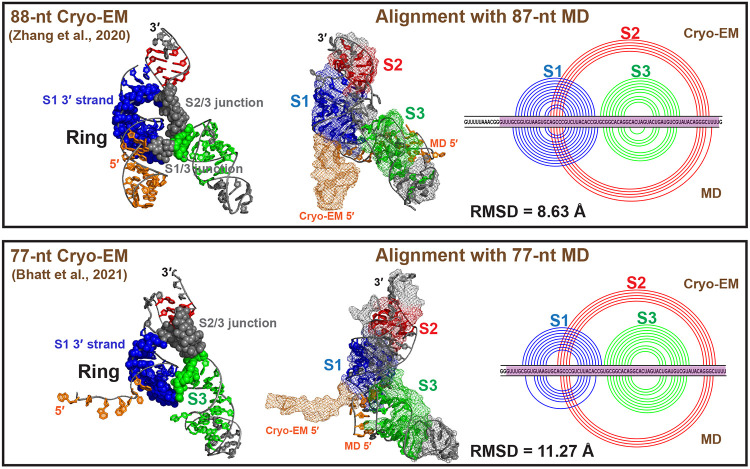
MD 3_6 structures compared to two Cryo-EM structures. (Top, Left) The 88-nt Cryo-EM structure^20^ in threaded ring conformation is (Middle) aligned with our 87-nt RNAComposer MD structure (Cryo-EM structure in mesh mode, MD in cartoon), with (Right) 2D structural comparisons (Cryo-EM arc plot at top, MD at bottom). The 3D structure alignment is performed by PyMol^48^ for 75 common residues (highlighted in purple in the 2D plot), and the RMSD is computed. (Bottom) Comparison between the 77-nt Cryo-EM structure^21^ and our 77-nt RNAComposer MD structure.

**Figure 6:**
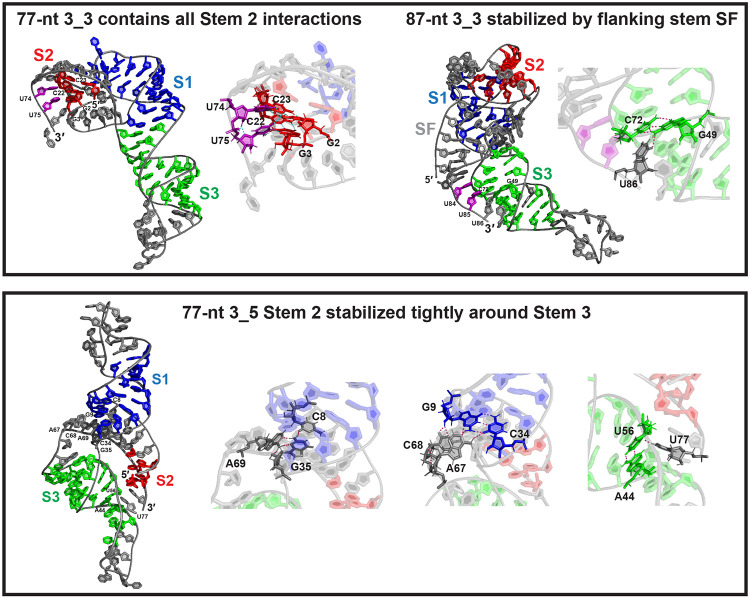
Alternative 3_3 and 3_5 conformations. (Top) The 77 and 87-nt 3_3 pseudoknot structures (iFoldRNA). For 77-nt, two residues in the 3′ end (purple), which are involved in the 3_6 and 3_5 Stem 2, form triplets with the 3_3 Stem 2. For 87-nt, the 5′ and 3′ end bind to form flanking stem SF, and the same two 3′ end residues (purple) are locked around Stem 3 by a downstream triplet. (Bottom) The 3_5 junction 77-nt MD model (SimRNA). The Stem 2 helix formed by the 5′ and 3′ ends is stabilized around Stem 3 by multiple hydrogen bonds.

**Figure 7:**
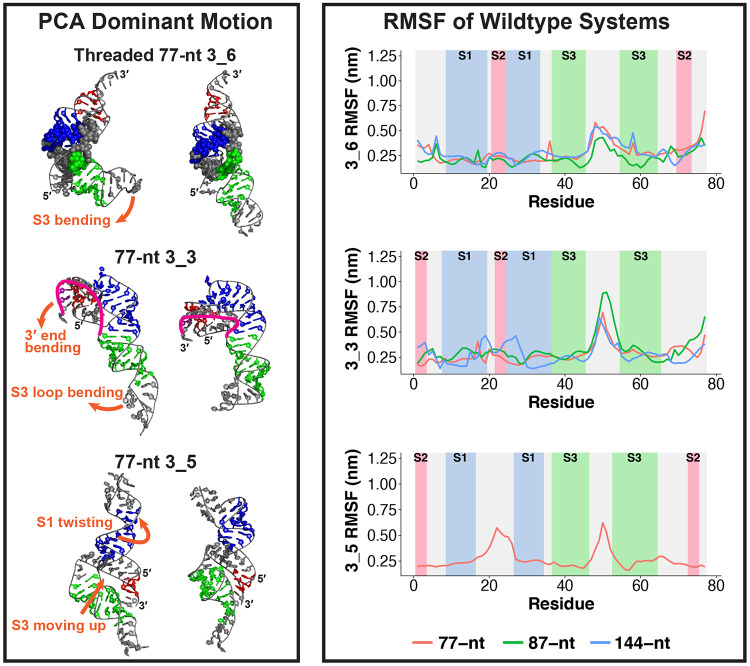
Dynamic analysis of the wildtype 3_6, 3_3, and 3_5 systems. (Left) Dominant motions of the threaded 77-nt 3_6 pseudoknot (RNAComposer), 77-nt 3_3 pseudoknot (iFoldRNA), and 77-nt 3_5 junction (SimRNA) extracted by principal component analysis (PCA). (Right) Flexibility of the three conformations as reflected by root mean square fluctuations (RMSF). For the 3_6 and 3_3 pseudoknots, the RMSF is shown for the common 77-nt region at various lengths; for 3_5 junction, RMSF at 77-nt. The different stem regions are colored and labeled.

**Figure 8:**
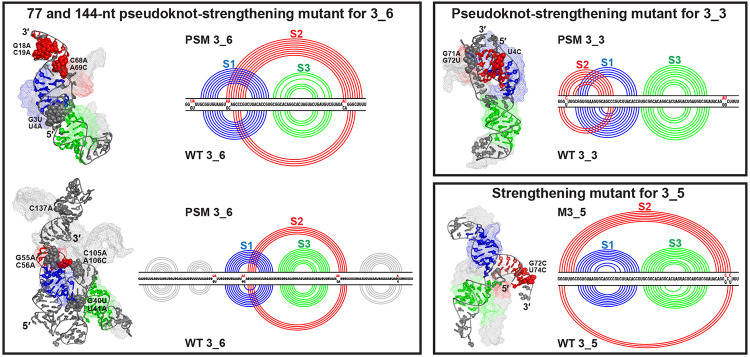
Comparison of the motif-strengthening mutants with the wildtype systems. For 3_6 pseudoknot, both the 77-nt (SimRNA) and 144-nt (RNAComposer) PSM are shown in cartoon mode with their wildtype systems aligned in mesh (by PyMol^48^ for the 77-nt region). The mutations are highlighted as spheres in PSM structure and labeled. The 2D structure comparison is also provided with PSM at top and wildtype at bottom. Comparisons for the 77-nt 3_3 PSM (iFoldRNA) and 77-nt 3_5 mutant (SimRNA) are shown in similar manner.

**Figure 9:**
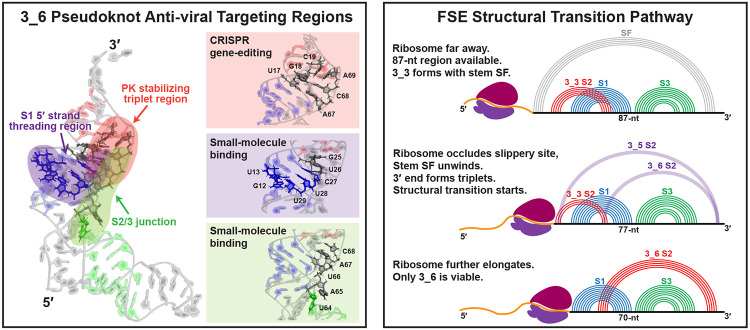
Implications of the unraveled structures and motions to anti-viral therapeutics and frameshifting mechanisms. (Left) Anti-viral target regions in the 3_6 pseudoknot. (Right) Proposed structural transition pathway for the SARS-CoV-2 frameshifting element.

Below, we analyze structures and motions obtained from microsecond molecular dynamics simulations for each conformation at different lengths. For the 3_6 pseudoknot, we identify critical tertiary interactions, such as hydrogen-bond networks that stabilize the pseudoknot complex (Fig. 3) and 5′ end threading that may be associated with ribosomal pausing (Fig. 4), and compare our structures with the Cryo-EM models (Fig. 5).^20,21^ For the alternative 3_3 pseudoknot and 3_5 junction, we discuss length-dependent flanking stem or triplet formation (3_3) and the Stem 2/3 interactions (3_5) that provide insights into FSE transitions (Fig. 6). Inherent motions of the three conformations and motif-strengthening mutants are discussed in Fig. 7 and 8. Notably, a key structural switch between the “L” and the linear shape for 3_6 that may send frameshifting signals to the ribosome is absent in the mutant. Global contraction and stretching in the 3_3 pseudoknot, along with triplet interactions, may play a role in frameshifting structural transitions. The combined insights suggest target regions for small-molecule binding and CRISPR gene-editing, as well as a structural transition pathway (Fig. 9).

### Tertiary interactions stabilize the 3_6 pseudoknot ring-like conformation

Throughout the microsecond MD simulations, the 3_6 pseudoknot motif is retained in all systems (Fig. S2). The shorter 77-nt structures adopt the “L” shape seen in the Cryo-EM studies^20,21^ (Fig. 3), with smaller radii of gyration (Rg) for systems more bent (Fig. S3), yet the total RNA potential energy is about the same for all (Fig. S4). The 87 and 144-nt structures become more linear, with vertical arrangement of the three stems (Fig. 3), similar to the recent crystallographic structure.^23^ The Rg and RNA potential energy increase slightly for 87-nt, while significantly for 144-nt by ~50% and ~200%, respectively.

Multiple hydrogen bonds act to stabilize the 3_6 pseudoknot complex (Fig. 3). In the 77-nt “L” shape, unpaired residues in the Stem 1/2 and 2/3 junctions form a quadruplet (U17, U26, U66, A67) and a triplet (G18, G25, C68) that define a short triplex, which is further extended by the binding of junction residue C19 with the 5′ helix end of Stem 2 (C24-G70). This triplex stabilizes the loose junctions and links the 3′ end tightly near the Stem 1 loop to maintain the pseudoknot. In the 144-nt linear shape, similar triplets are formed by the 3′ helix end of Stem 2 and the downstream loop residues to seal the short Stem 2.

The ring in all 3_6 systems forms by linking the 3′ strand of Stem 1, Stem 1/3 junction, 5′ helix end of Stem 3, and Stem 2/3 junction (Fig. 4). In a recent 88-nt Cryo-EM structure (6.9 Å resolution), the 5′ end is reported to thread through the ring.^20^ Here, we capture both threaded and non-threaded ring conformations at various sequence lengths (Fig. 4). For 77-nt, the 5′ strand of Stem 1 and the 5′ end can either thread through the ring (“threaded”) — possibly hampering ribosomal unwinding and promoting longer ribosomal pausing — or wind around the structure (“non-threaded”). For 87-nt, the ring holes are larger, probably due to longer Stem 1, and the extended FSE 5′ end winds around Stem 3 in both threaded and non-threaded structures, though in opposite orientations.

We also identify ring-stabilizing hydrogen-bond networks (Fig. 4). For threaded 77-nt, the Stem 1 loop (U17, U26) and the Stem 2/3 junction (A66, A67) bind to seal the ring top. For threaded 87-nt, a similar triplet (C29, G35, A79) forms at the ring top, and two more at the ring bottom by the 5′ helix end of Stem 1 (C18-G45, G17-U46) with the Stem 1/3 and 2/3 junctions (G47, U74). In non-threaded systems, triplets form at both the ring top by the 3′ helix end of Stem 1 and junctions, and the ring bottom by the 5′ helix ends of Stems 1 and 3.

Comparing our 87-nt threaded 3_6 to the 88-nt Cryo-EM structure (6.9 Å resolution),^20^ and our 77-nt threaded 3_6 to the 77-nt Cryo-EM mRNA-ribosome complex (5-7 Å),^21^ we find that the experimental “L” shape with coaxially stacked Stems 1 and 2 are globally similar to our MD structures (Fig. 5). Our models have narrowed ring holes and shorter Stem 3. In the mRNA-ribosome complex, Stem 3 bends more towards the S1/S2 coaxial plane. The 5′ end shows more differences: the 5′ end of the 88-nt Cryo-EM structure forms a small stem-loop, while our 5′ end loosely winds around Stem 3; the 5′ end of the mRNA-ribosome complex is pulled outward, likely explained by the presence of ribosomal interactions. Overall, our independently developed yet well aligned 3_6 MD structures provide credibility for the following alternative structure modeling.

### Alternative 3_3 pseudoknot and 3_5 junction provide insights on structural transitions

The alternative 3_3 pseudoknot, dominant in our 87 and 144-nt FSE constructs,^3^ contains a different Stem 2 formed by the Stem 1 loop and the 5′ end. At 77-nt, the 3_3 pseudoknot has a short Stem 2 (3 base pairs); at 87 and 144-nt, upstream residues form 2 additional base pairs for Stem 2, and also a flanking stem SF with the 3′ end to further seal the conformation (Fig. 6, more details in Fig. S5, S6). Likely due to SF, the 87-nt 3_3 structures have smaller Rg than 77-nt, and they are much more compact than 3_6 (Fig. S3).

The length-dependent interactions in the 3_3 trajectories suggest a potential intermediate mRNA structure that facilitates structural transitions during ribosomal translation and RNA refolding. For 77-nt, the 3′ end residues U74 and U75 form two triplets with two 3_3 Stem 2 base pairs G2-C23 and G3-C22 (see Fig. 6). In 3_6, the same end residues U74, U75 base pair with A20 to form Stem 2; in 3_5, they pair with G2 and G1 to form Stem 2. Hence, all three Stem 2 interactions co-exist in this 77-nt 3_3 structure, and this state suggests a starting conformation for a structural transition from 3_3 to 3_6 or 3_5.

For the 87-nt 3_3 systems, the flanking stem SF by the 5′ and 3′ ends blocks alternative Stem 2, and the hydrogen bonding between residue U86 and the Stem 3 base pair C72-G49 maintains the 3′ end away from Stem 2 (Fig. 6). In our 144-nt models, additional stems form to avoid the mixed Stem 2 triplets (Fig. S5, S6). Hence, all these interactions, especially stem SF must be unwound by the ribosome before the 3′ end is free to form alternative 3_6 Stem 2 (with the Stem 1 loop) or 3_5 Stem 2 (with the 5′ end).

Our 3_5 3-way junction RNA at 77-nt is retained in all trajectories (Fig. S7), where the 5′ and 3′ ends base pair to form Stem 2. This motif has similar Rg and RNA potential energy to the other two conformations at 77-nt (Fig. S3, S4). A typical 3_5 conformation is elongated in shape as shown in Fig. 6, with Stems 1 and 2 coaxially stacked.

Interactions that impede structural transitions also exist in the 3_5 structure. The 3′ end residue U77 hydrogen bonds with Stem 3 base pair A44-U56 to keep Stem 2 near Stem 3 (Fig. 6). Moreover, the Stem 1/3 junction, the 5′ helix end of Stem 1, and the Stem 1/2 junction form a triplet and a quadruplet to further lock the Stem 2 orientation and avoid alternative Stem 2. Similar to the 87-nt 3_3, these hydrogen bonds must be broken to allow transition to another motif.

### Fluctuations and dominant motions of the three conformations

Using principal component analysis (PCA), we capture the dominant motion for 3_6 to be a structural switch between the “L” and the linear shape, via bending of Stem 3 (Fig. 7). The pseudoknot complex (Stems 1 and 2) and the ring conformation are maintained throughout this motion, as does the ring-holding triplet at the bottom. Longer 3_6 structures tend to remain linear, with upstream and downstream stems moving more substantially (Fig. S8).

Consistent with the above motions, we see a peak in the 3_6 root mean square fluctuations (RMSF) in the Stem 3 loop region for all lengths (Fig. 7). The unpaired 3′ end also exhibits high RMSF, especially for 77-nt, as no downstream pairs restrict its movement. The RMSF, average number of hydrogen bonds (H-bond), and the interaction energies all indicate that Stem 1 is the strongest, followed by Stem 3, and lastly by Stem 2 (Fig. S9, S10).

The 3_3 pseudoknot′s dominant motion is contraction and stretching caused by the bending of 3′ end and Stem 3 loop (Fig. 7, Fig. S11). In this motion, Stems 1 and 2, especially triplets that contain interactions from all three Stem 2 (purple and red residues in Fig. 7), are stable and move in unison. That these triplets are not transient suggests that they may be part of the structural transition among alternative conformations, as discussed above.

Comparing to 3_6, we see a higher RMSF peak value in the 3_3 Stem 3 loop region, and more fluctuations in 3_3 Stem 1 region due to the pseudoknot bending, with a consistent lower Stem 1 H-bond number (Fig. S9). A clear jump occurs for 3_3 Stem 2 H-bond number, when the length increases from 77 to 87-nt, resulting in a stronger Stem 2 of 3_3 than 3_6 (Fig. S9), following our finding of dominant 3_6 at 77-nt while dominant 3_3 at 87-nt.^3^ A similar trend is observed for the stem interaction energy (Fig. S10).

For the 3_5 junction, Stem 1 twisting is dominant (Fig. 7, Fig. S12): as Stem 1 twists backwards, it pulls the downstream backbone and hence Stem 3 moves up towards Stem 1. All the triplets and hydrogen bonds that lock the Stem 2 orientation (Fig. 6) are maintained, and Stem 2 is kept near Stem 3 while they move together. Peak RMSF in the loop regions of Stems 1 and 3, and low values in the 5′ and 3′ ends are notable.

Overall, all three conformations have stable Stem 1, flexible Stem 3 loop, and relatively stable Stem 2 regions. The triplets and hydrogen bonds are mostly maintained throughout the simulations, and this helps stabilize key features such as the ring of 3_6 and the combined Stem 2 interactions in 3_3.

### Minimal mutations stabilize the three conformations

Our predicted mutations confirmed by SHAPE probing were designed to suppress conformational transitions and stabilize specific conformations over all alternatives, for the 77 and 144-nt 3_6 pseudoknot, 77-nt 3_3 pseudoknot, and 77-nt 3_5 junction.^3,25^ Our dynamics analyses below of these mutants compared to the wildtype trajectories help interrogate the mechanisms and consequences of structural stability; we use the same representative mutant systems in Fig. 8 as for the wildtype, except for 77-nt 3_6.

The 6 mutations in the 77-nt 3_6 pseudoknot-strengthening mutant (PSM) include 4 mutations ([G18A, C19A, C68A, A69C]) that lengthen Stem 2 by up to 4 base pairs (Table 1) and 2 mutations at the 5′ end to exclude alternative 3_3 and 3_5 Stem 2. Because the SimRNA mutant has the longest Stem 2 (9 base pairs), we compare it to the corresponding wildtype. We observe a dramatic transformation from “L” shape (wildtype) to a linear shape (Fig. 8). Indeed, all 3_6 mutant systems adopt this linear shape, and the structural switch between the two shapes has been suppressed (Fig. S13, S14).

For the 144-nt 3_6 PSM, one additional mutation in the downstream region suppresses formation of competing stems.^3^ The central 3_6 pseudoknot region aligns well between the wildtype and mutant systems, both adopting the linear shape (Fig. 8, Fig. S13). The major difference occurs in the upstream region: in the wildtype, upstream and downstream stems form on the same side of the central 3_6 pseudoknot; in the mutant, they are on different sides, due to our [G40U, U41A] mutations. From PCA, we see a relatively stable central 3_6 pseudoknot, while quite flexible upstream and downstream stems in the mutant (Fig. S14). As both our 77 and 144-nt 3_6 mutants adopt linear conformations, we hypothesize that this may be a more stable conformation, by separating the 5′ and 3′ ends further away from each other to avoid alternative 3_3 and 3_5 Stem 2.

In our 77-nt 3_3 PSM, a large increase of Stem 2 length from 3 to 7 base pairs is induced by mere three mutations [U4C, G71A, G72U] (Table 1, Fig. S15). The first mutation enhances the 3_3 Stem 2 and the others avoid alternative 3_6 and 3_5 motifs. The main structural changes are a vertical 5′ end between the Stem 1 loop and helix instead of staying horizontal below, compact Stems 1 and 2, and elimination of triplets formed by the 3′ end with Stem 2 (Fig. 8). Hence, our mutations stabilize the 3_3 conformation without alternative Stem 2 interactions. The dominant motion occurs in the Stem 3 region (Fig. S16).

Our 77-nt 3_5 mutant with only 2 mutations [G72C, U74C] also enjoys a considerable enhancement of Stem 2 from 3-4 base pairs to 6-7 (Table 1, Fig. S17). The three stems then have similar sizes (Fig. 8). Stem 2 is no longer held around Stem 3, but instead extends as a third helical arm. Coaxial stacking of Stems 1 and 2, as well as a tilting motion of these two stacked stems, are observed (Fig. S18).

Overall, our enhanced Stem 2 in the three mutants leads to dramatic structural changes, especially for the 77-nt 3_6 and 3_5 systems. PCA analysis reveals stabilization of the linear shape in 3_6 PSM, thereby eliminating the “L” to linear shape switch. For the 77-nt 3_3 mutant, triplets associated with possible structural transitions are also eliminated.

## Discussion: Implications to frameshifting and anti-viral strategies

Our microsecond MD simulations of three possible conformations of the SARS-CoV-2 FSE, namely 3_6 pseudoknot, 3_3 pseudoknot, and 3_5 junction for different lengths (Fig. 1, 2), highlight different structural features and motions. Our motif-strengthening mutant trajectories clarify how these mutations alter the RNA conformations and motions (Fig. 8). The combined insights suggest three anti-viral intervention avenues and a mechanism for frameshifting that links our three alternative conformations (Fig. 9).

The first anti-viral approach is to alter the 3_6 pseudoknot plasticity. Pseudoknot stabilizing hydrogen bonds are identified at Stem 1/2 and 2/3 junctions of 3_6 (Fig. 3). Since conformational plasticity has a large impact on frameshifting efficiency,^9^ mutating these residues to further strengthen or destroy the pseudoknot should interrupt the frameshifting process. Indeed, Bhatt et al. achieve a significant reduction in frameshifting efficiency by mutating these junctions.^21^ In our prior SHAPE probing, 3_6 Stem 2 enhancing mutations in this region modify the conformational landscape to 100% 3_6.^3^ Both studies underscore the sensitivity of the 3_6 pseudoknot and its associated frameshifting to these junction residues, which define good targets for CRISPR gene-editing (Fig. 9, left).

The second approach is to strengthen the 5′ end threading in the 3_6 ring conformation. The ring is formed by the 3′ strand of Stem 1, the Stem 1/3 and 2/3 junctions, and are stabilized by hydrogen bonding and base triplet interactions (Fig. 4). In some systems, the 5′ strand of Stem 1 and the 5′ end thread through the ring, which probably resists ribosomal unwinding^20^ by requiring a higher unfolding force;^49^ thus, strengthening the threading may increase the mechanical barrier for translation. Recently, two *alkaloids* (*emetine* and *cephaeline*) predicted to bind the threading initiation site were found to inhibit SARS-CoV-2 viral replication.^50^ Hence, the 3′ helix end of Stem 1, which we find to close the ring and initiate threading, defines a target binding region to impede ribosomal translation (Fig. 9).

The third approach is to target the 3_6 pseudoknot structural switch between an “L” shape (coaxially stacked Stems 1 and 2 and an extruding Stem 3) and a linear shape (vertical stacking of the 3 stems), revealed by our PCA analysis (Fig. 7). In the mRNA-ribosome Cryo-EM structure captured during translation,^21^ the “L” shaped 3_6 wedges at the mRNA entry channel and resists unwinding by the helicase, which generates tension on the upstream mRNA.^21^ This structural switch might then enhance fluctuations of this tension and send frameshifting signals to the ribosome. When switching from the “L” to linear shape, residues in the Stem 2/3 junction are exposed (Fig. 7); small molecules like *MTDB*^10,51^ can thus block the switch and hamper frameshifting (Fig. 9). Another option is to deploy our 3_6 mutant, which assures a stabilized linear shape (Fig. 8).

Overall, by analyzing the hydrogen bonding interactions and motions of different 3_6 systems, we offer three strategic anti-viral targeting regions: the 3′ helix end of Stem 1 and Stem 1/2 and 2/3 junction residues (Fig. 9). Although several drugs/small molecules have been shown to inhibit SARS-CoV-2 frameshifting, including *MTDB*,^18,51,52^ *alkaloids*,^50^ *Merafloxacin*,^53^ *Ivacaftor*, and *Huperzine A*,^54^ they are mainly found by high-throughput drug screening, so the underlying inhibition mechanism is unexplained and, in some cases, the binding regions are unknown. Our targeting regions above emerged from mechanistic considerations.

Furthermore, based on interactions analyzed in our trajectories of different lengths (Fig. 6), we propose a possible FSE structural transition pathway (Fig. 9, right): during translation, when the ribosome is far away from the FSE region, the dominant conformation is a 3_3 with stem SF; as the ribosome approaches and occludes the slippery site, stem SF is unwound, and the 3′ end moves to the 3_3 Stem 2 region to form the triplets and structural transition to 3_6 or 3_5 begins; when the ribosome further elongates, the 5′ end (including the slippery site) becomes completely occluded, and only 3_6 remains viable.

This structural transition pathway may be associated with SARS-CoV-2 regulatory functions, as RNA structural alterations can lead to different biological outcomes.^55^ For example, ribosomal RNA (rRNA) samples alternate structures to control translation.^56^ The timescale at which the transitions occur depends on the scale of conformational rearrangements. Interhelical or loop dynamics occur on picosecond to microsecond timescale. Base pairing or tertiary structure changes occur on microsecond to second range. Major interconversions between secondary structures occur on millisecond and longer.^57^ Given that the ribosome pauses ~2.8s between translocations,^58^ this time allows for the structural switches and transitions discussed here to occur.

In sum, our microsecond MD simulations extend beyond consistent 3D structure models for the prevalent 3_6 pseudoknot in literature,^3,17-22,24,25,38,59^ by providing the first 3D models for the alternative FSE structures and the motif-strengthening mutants, which were verified by SHAPE experiments.^3^ We suggest several potential interventions to interfere with SARS-CoV-2 frameshifting and ribosomal translation, and provide insights into frameshifting mechanism (Fig. 9). These ideas offer anti-viral strategies against Covid-19 by small-molecule binding and CRISPR gene-editing. More broadly, our methods and analyses extend to other viral systems. Together with other computational and experimental studies, we hope to advance our understanding of the basic science associated with complex frameshifting mechanisms and therapeutic applications.

## Materials and Methods

### RAG Notation and Mutations

In our RNA-As-Graphs (RAG) framework, RNA secondary structures containing pseudoknots are represented as dual graphs.^27^ Each stem (≥ 2 base pairs) denotes a vertex, and every single strand or loop is an edge (hairpins are self-loops; 1-nt bulges, internal loops with two 1-nt strands, and dangling ends are ignored). Every non-isomorphic dual graph is assigned an identifier *V_n*, where *V* is the vertex number and *n* is a unique motif identifier. Our dual graph library consists of over 100,000 unique dual graphs for 2-9 vertices.^30^

To design RNAs with minimal mutations that make the FSE fold *in silico* onto a target dual graph, we developed our inverse folding program RAG-IF modified for dual graphs.^25,36^ For manually selected mutation regions and a target 2D structure, RAG-IF uses a genetic algorithm to generate a pool of candidate RNA sequences with mutations. These candidates are screened by 2D prediction programs to ensure the correct graph folding, and are optimized for minimal mutations. Detailed design of the mutants is described in.^3,25^

### FSE Lengths and Conformations

We model the FSE structure at three sequence lengths: 77-nt without the 7-nt slippery site, 87-nt with the slippery site plus 3 additional residues at the 5′ end, and 144-nt with the slippery site plus 30 additional residues at each end. We perform MD simulations for all three conformations for the 77-nt FSE. (Even though the 3_3 pseudoknot was not observed at this length, we study it for comparison with other lengths.) For 87 and 144-nt, we model the 3_6 and the 3_3 conformations, with additional stems formed by the upstream and downstream nucleotides (Fig. 1).

Besides wildtype FSEs, we also model four motif-strengthening mutants predicted previously:^3,25^ 77-nt 3_6 PSM with 6 mutations [G3U, U4A, G18A, C19A, C68A, A69C], 144-nt 3_6 PSM with an additional mutation C137A, 77-nt 3_3 PSM with 3 mutations [U4C, G71A, G72U], and 77-nt 3_5 mutant with 2 mutations [G72C, U74C].

### 2D and 3D FSE Structures

The 2D structure of the wildtype 77-nt 3_6 pseudoknot was predicted by PKNOTS,^60^ and all other 2D conformations were modeled by ShapeKnots with SHAPE reactivities incorporated.^3,61^

Corresponding 3D structures were predicted, with the sequences and the 2D structures as input using RNAComposer,^62^ Vfold3D,^63^ SimRNA,^64^ and iFoldRNA^65^ for 77 and 87-nt, and RNAComposer, iFoldRNA, and Farfar2^66^ for 144-nt, as SimRNA and Vfold3D failed to produce models for this length (see Table S1). For 3D structure prediction programs that gave multiple structures as output, the first structure that retained the correct motif was selected for MD simulations.

### Molecular Dynamics Details

The MD simulation protocol follows our prior work.^25^ We use Gromacs 2020.3 and 2020.4,^47^ with the Amber OL3 forcefield.^67^ The systems are solvated in the cubic box with TIP3P water model, with a buffer of 10 Å from the RNA molecule.^68^ The systems are first neutralized with sodium ions and set to a 0.1M NaCl bulk concentration with additional Na^+^ and Cl^−^ ions. The systems are energy minimized via steepest descent and equilibrated under NVT (300 K) and NPT (1 bar and 300 K) ensembles for 100 ps each. Simulations are run with a timestep of 2 fs and a SHAKE-like LINCS algorithm^69^ with constraints on all atom bonds. The Particle Mesh Ewald method^70^ is used to treat long-range electrostatics. Production runs are performed for 1~1.5 *μ*s under NPT to ensure stable RMSD. Structures from the last 500 ns of each simulation are used for analysis.

Clustering is performed on frames every 200 ps for RNA non-H backbone atoms, using the Gromos clustering method with 2, 2.5, 3, and 3.5 Å cutoffs. The largest cluster center structures (cutoff of 2.5Å for 77-nt and 87-nt systems or 3.5Å for 144-nt systems) are extracted from MD simulations to show and analyze in Results and Supplementary Information. The cutoffs are chosen to ensure that all simulations for the same dual graph topology produce a feasible number of clusters with outlier structures excluded. See Fig. S19 for more details.

PCA is performed on structures every 250 ps. Cluster analysis, PCA motion analysis, calculations of Rg, RMSF, RNA potential energy, interaction energy (sum of short-term Lennard-Jones and Coulomb interactions) between the two strands within each stem, and the number of hydrogen bonds in each stem are performed via Gromacs 2020.3.^47^ The 2D structures, base pairing, and stacking information are analyzed using 3DNA-DSSR.^46^ The structure alignment is performed using PyMol^48^ *align* with RMSD computed.

All microsecond MD simulations were conducted on the Prince or Greene supercomputer clusters at the New York University High Performance Computing facilities. Each compute node in the Prince cluster is equipped with two Intel Xeon E5-2690v4 2.6 GHz CPUs (“Broadwell,” 14 cores/socket, 28 cores/node) and 125 GB memory. Each simulation is performed with seven to eight dedicated nodes (i.e., 196–224 cores), so the simulations complete in 7–10 days. Each compute node in the Greene clusters is equipped with two Intel Xeon Platinum 8268 24C 205W 2.9GHz CPUs with 48 cores/node and 192 GB memory. Each simulation is performed with 30 nodes using 32 cores each, so that the simulations complete in 2-4 days.

## Supplementary Material

Supplement 1

## Figures and Tables

**Table 1:** Comparison of the motif-strengthening mutants and the wildtype systems. For each mutant, the mutations, the 3D prediction programs (*R* for RNAComposer, *S* for SimRNA, *I* for iFoldRNA, *V* for Vfold3D), the wildtype and mutant Stem 2 lengths, and the newly formed Stem 2 base pairs involving the mutated residues are listed.

Program	WT S2	Mutant S2	Base pairs involving mutations
**77-nt 3_6 PSM [G3U, U4A, G18A, C19A, C68A, A69C]**			
R	4	4	G25-C69
S	4	9	A18-U76, A19-U75, G25-C69, U26-A68
I	7	7	A19-U75, G25-C69
V	4	8	A18-U76, A19-U75, G25-C69, U26-A68
**144-nt 3_6 PSM [G40U, U41A, G55A, C56A, C105A, A106C, C137A]**			
R	4	5	G62-C106
I	5	4	A56-U112
**77-nt 3_3 PSM [U4C, G71A, G72U]**			
I	3	7	C4-G21
**77-nt 3_5 Mutant [G72C, U74C]**			
R	3	7	G1-C74, G3-C72
S	3	7	G1-C74, G3-C72
I	4	7	G1-C74, G3-C72
V	3	6	G3-C72
