# Supplementary material for: Length-dependent motions of SARS-CoV-2 frameshifting RNA pseudoknot and alternative conformations suggest avenues for frameshifting suppression
Source: Res Sq. 2022 Jan 4:rs.3.rs-1160075. Preprint. [Version 1] doi: 10.21203/rs.3.rs-1160075/v1 (PMC8750709; doi:10.21203/rs.3.rs-1160075/v1)
Supplement: Supplement 1 [file 507cc9e693876b171fe9430b.pdf]

Supplementary Information for Length-dependent motions of SARS-CoV-2 frameshifting RNA pseudoknot and alternative conformations suggest avenues for frameshifting suppression

Shuting Yan,[†,‡] Qiyao Zhu,[†,¶] Swati Jain,[‡] and Tamar Schlick[*,‡,¶,§]

†*These authors contributed equally to this work.*

‡*Department of Chemistry, New York University, New York, NY 10003 U.S.A.*

¶*Courant Institute of Mathematical Sciences, New York University, New York, NY 10012 U.S.A.*

§*NYU-ECNU Center for Computational Chemistry, NYU Shanghai, Shanghai 200062, P.R. China*

E-mail: schlick@nyu.edu

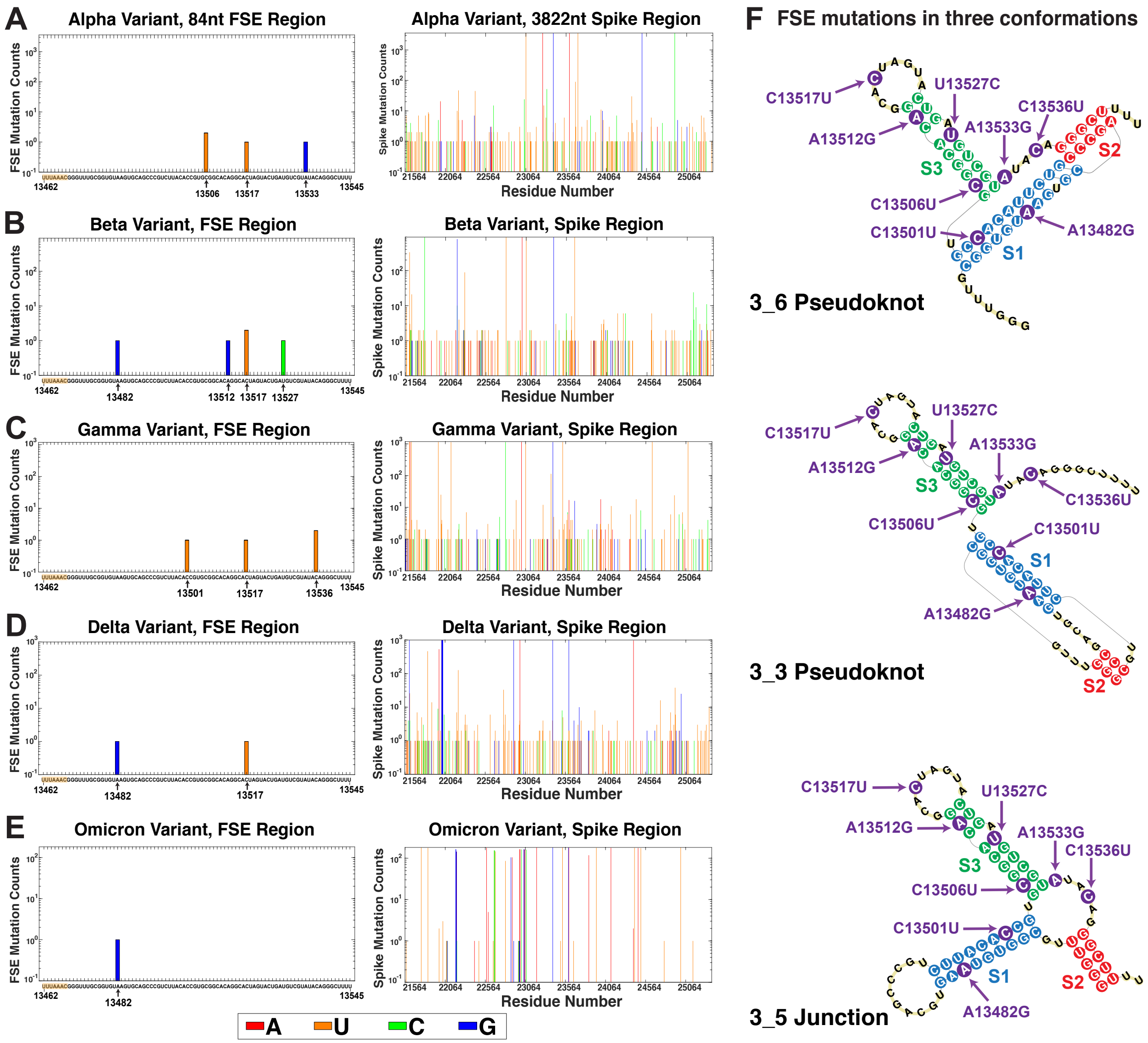

Figure S1: SARS-CoV-2 RNA mutation maps for the (A) Alpha variant, (B) Beta variant, (C) Gamma variant, (D) Delta variant, and (E) Omicron variant for the 84-nt FSE region and the spike gene region. (F) The mutations in the FSE region are labeled in the three conformations 3_6, 3_3, and 3_5.

We produce mutation maps for the 84-nt FSE region and the 3822-nt spike gene region of the five major COVID variants (Alpha, Beta, Gamma, Delta, and Omicron) (Fig. S1). 3575 available Alpha and 898 Beta variant RNA sequences are downloaded from GISAID[1] on February 8, 2021; 1170 Gamma and 1000 Delta sequences are randomly selected from those downloaded on July 8, 2021; 182 available Omicron sequences are downloaded on November 30, 2021. We then align them with the official SARS-CoV-2 RNA reference sequence of 29891-nt provided by GISAID (Accession ID: EPI_ISL_402124), following the same protocol used in our prior paper.[2] The FSE region occupies residues 13462–13545, and the spike gene region occupies 21564–25384.

The FSE region mostly has single-nucleotide mutation in $<1\%$ variant sequences (Fig. S1). For the Alpha variant, only 4 out of 3575 sequences have single mutations (C13506U twice, C13517U once, and A13533G once); for Beta and Gamma, only 5/898 and 4/1170, and besides the common C13517U mutation, we observe some new mutations (A13482G, A13512G, and U13527C for Beta, C13501U and C13536 for Gamma); for Delta and Omicron, only 2/1000 and 1/182, and no new mutations.

We label all the FSE mutations in the three conformations 3_6, 3_3, and 3_5 (Fig. S1). These mutations are either in Stems 1 and 3, or in the loop regions. They are all transition mutations, i.e., pyrimidine-pyrimidine or purine-purine, and as a result, the 2D structures would not be affected. For example, the A13482G mutation appeared in Beta, Delta, and Omicron variants locates in the $5'$ strand of Stem 1, forming an A-U base pair in the wildtype, so after mutation, it can still form a G-U wobble base pair.

On the other hand, the spike gene can have dozens of mutations per sequence, and new mutations are added every time when a new variant appears. All Alpha sequences have 5-11 mutations in the spike gene, and there are 7 high frequency mutations that occur in $>70\%$ of the sequences; for Beta, 4-12 mutations per sequence, and 7 high frequency mutations; for Gamma, 2-25 mutations per sequence, and 12 high frequency mutations. Noticeably, these high frequency mutations are mostly different for each variant. More mutations emerge since the Delta variant, having 8-24 mutations per sequence, and 18 high frequency mutations. For Omicron, 15-45 mutations are possible per sequence, and out of 36 high frequency mutations, only 5 have been seen in the previous variants.

Therefore, as the spike gene region keeps adding new mutations that change the translated protein structures, the FSE region has very few mutations, even for the Delta and Omicron variants. Moreover, the FSE mutations stay in the same set for all the variants, without introducing new ones, and they seem to maintain the FSE conformation by forming alternative Wobble base pairs or mutating the loop regions. This high conservation of FSE then makes it a good drug target.

Table S1: FSE systems studied in this work for three conformations 3_6, 3_3, and 3_5 and their motif-strengthening mutants (denoted by M) at different lengths, using several 3D prediction programs (*R* for RNAComposer, *S* for Sim-RNA, *I* for iFoldRNA, *V* for Vfold3D, and *F* for Farfar2).

Length	WT 3_6	WT 3_3	WT 3_5	M 3_6	M 3_3	M 3_5
77nt	R, S, I, V	R, S, I, V	R, S, I, V	R, S, I, V	R, I	R, S, I, V
87nt	R, S, I, V	R, S, I, V	N/A	N/A	N/A	N/A
144nt	R, I, F	R, I, F	N/A	R, S, I	N/A	N/A

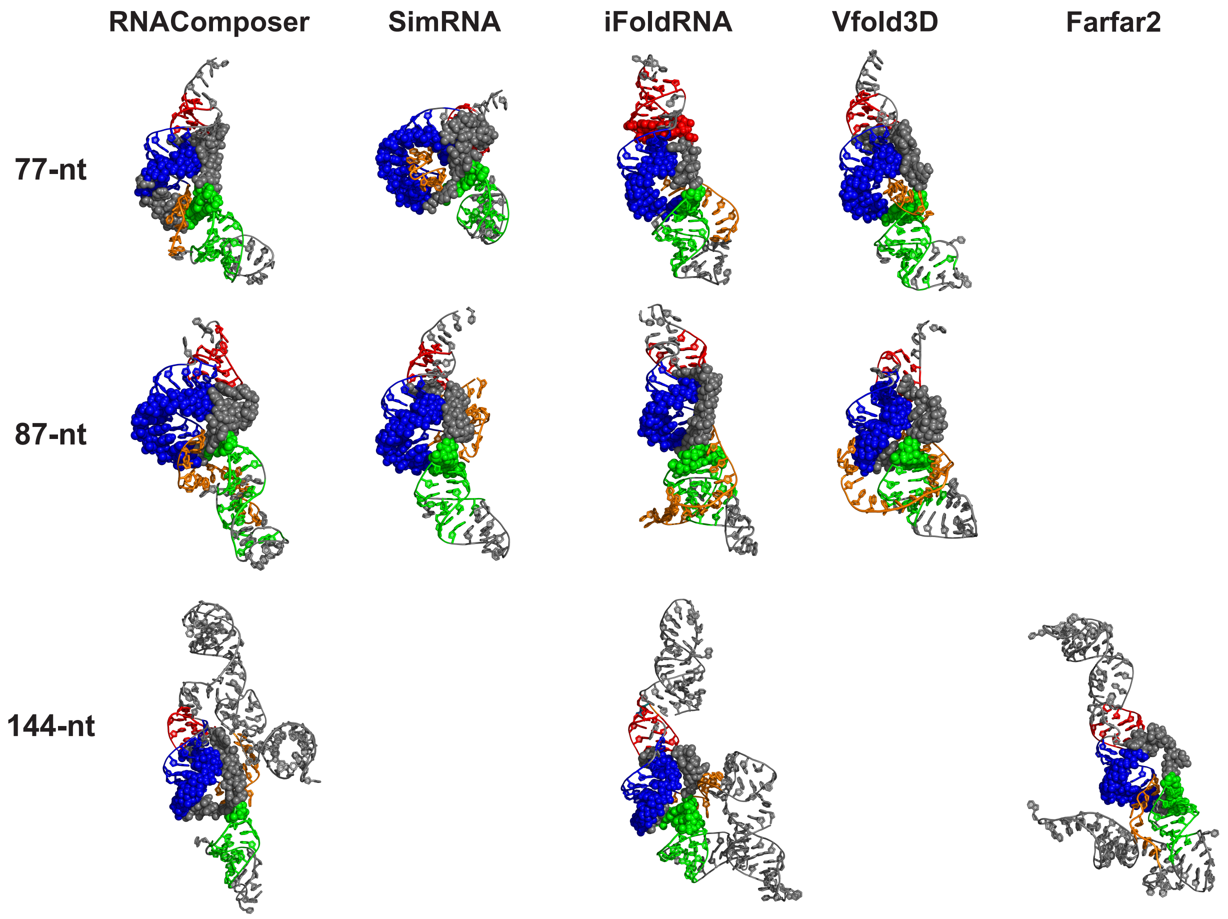

Figure S2: Largest cluster center structures of wildtype 3_6 pseudoknot for lengths 77, 87, and 144-nt using different 3D structure prediction programs. Stem 1 is colored blue, Stem 2 red, and Stem 3 green. The ring formed by the 3′ strand of Stem 1, the Stem 2/3 junction, and the Stem 1/3 junction is highlighted. The 5′ strand of Stem 1 and the 5′ end are colored orange, and in some systems, they thread through the ring.

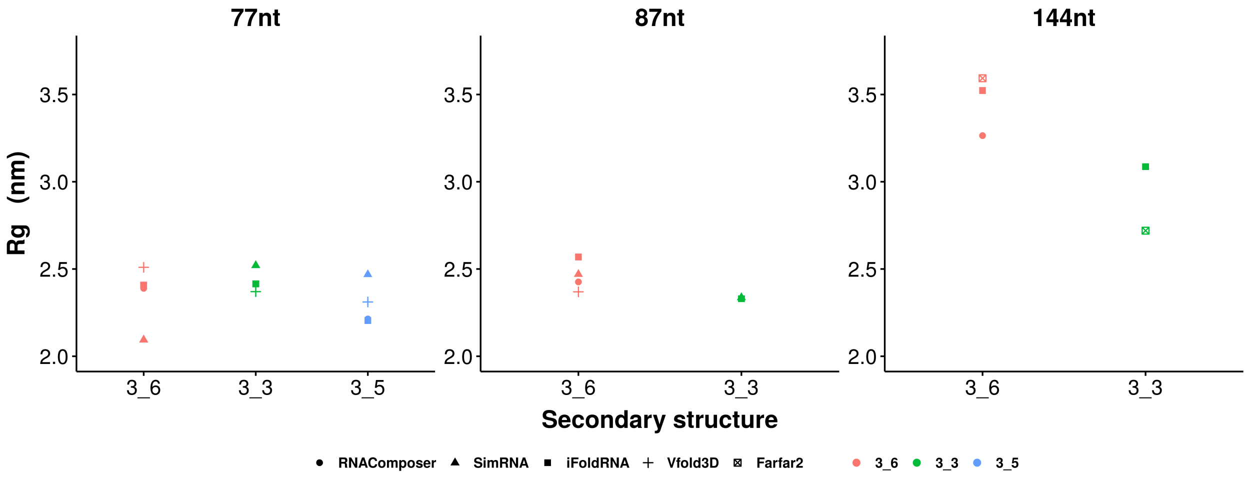

Figure S3: Radius of gyration of the wildtype 3_6, 3_3, and 3_5 conformations for 77, 87, and 144-nt. The 3_3 pseudoknot is more compact in longer sequences than 3_6.

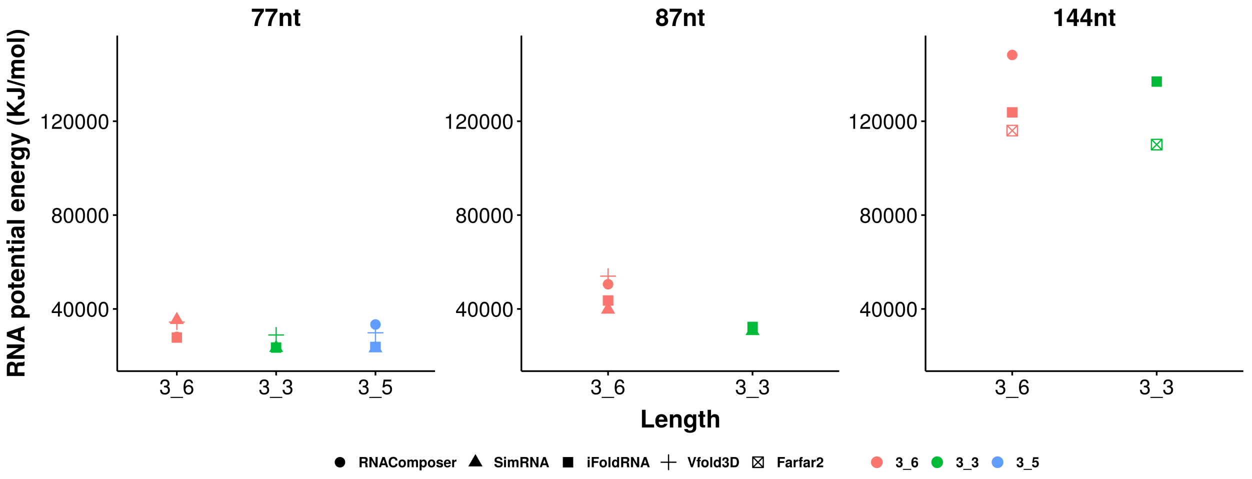

Figure S4: RNA potential energy of the wildtype 3_6, 3_3, and 3_5 conformations for 77, 87, and 144-nt. The 3_3 pseudoknot shows lower RNA potential energy than 3_6 in 87 and 144-nt FSE.

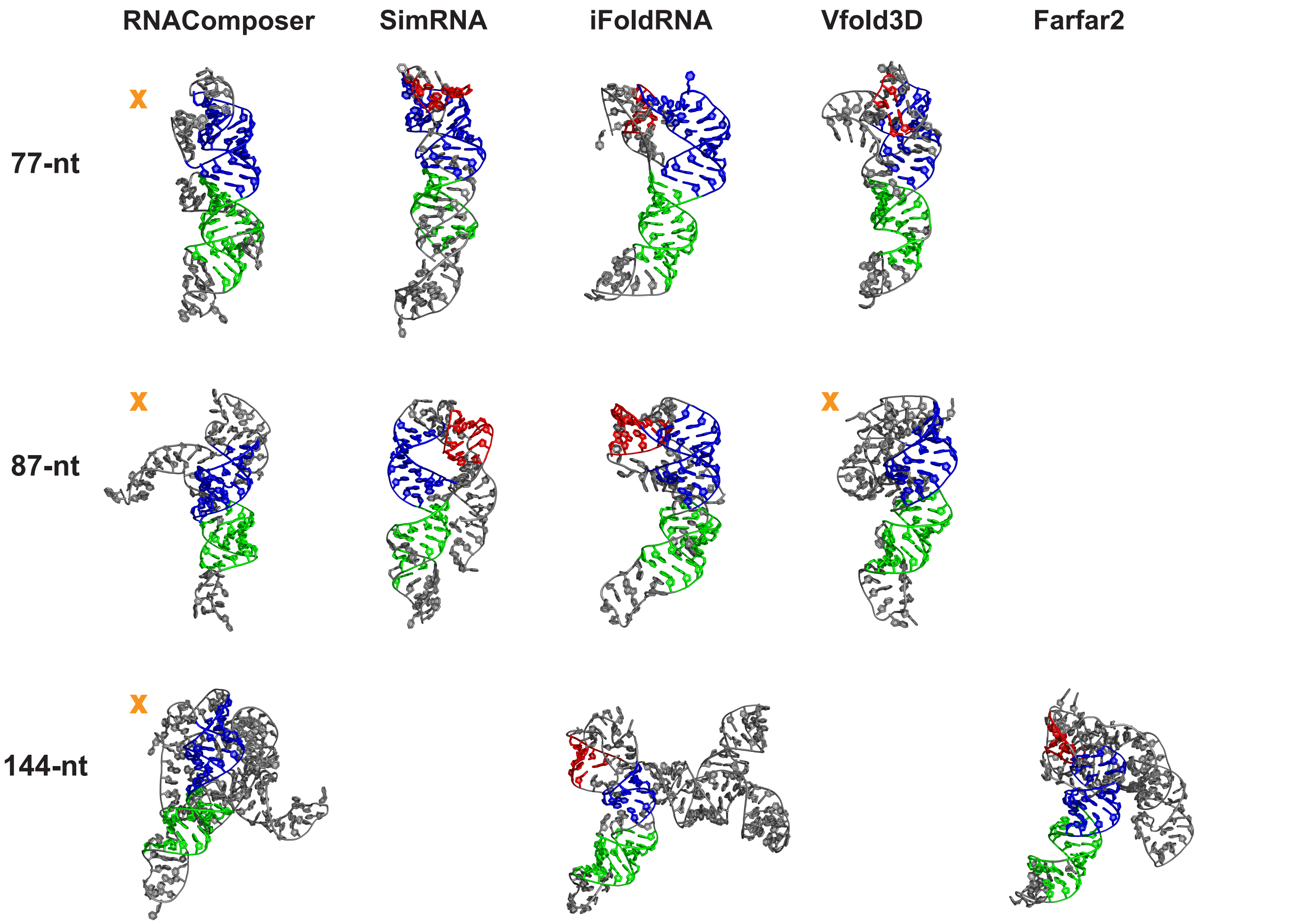

Figure S5: Largest cluster center structures of wildtype 3_3 pseudoknot for lengths 77, 87, and 144-nt using different 3D structure prediction programs. Stem 1 is colored blue, Stem 2 red, and Stem 3 green. The 77-nt RNAComposer, the 87-nt RNAComposer and Vfold3D, and the 144-nt RNAComposer systems fail to form 3_3 Stem 2, and they are marked by an "x".

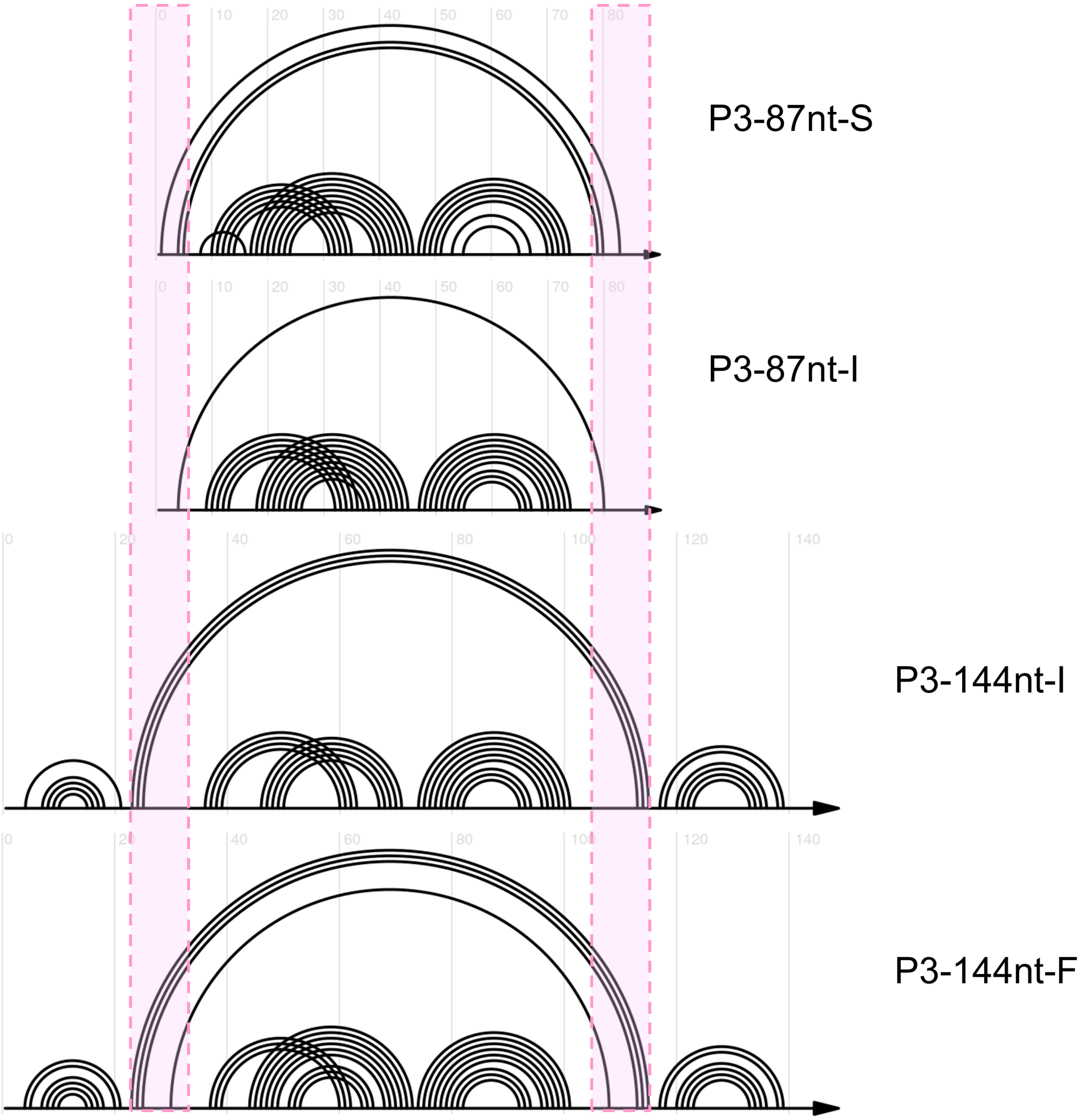

Figure S6: Secondary structures of the 3_3 pseudoknot for 87 and 144-nt FSE. Canonical Watson-Crick and wobble base pairs are shown for the largest cluster center structures from microsecond MD simulations. The pink box represents flanking stem SF formed by upstream and downstream sequence.

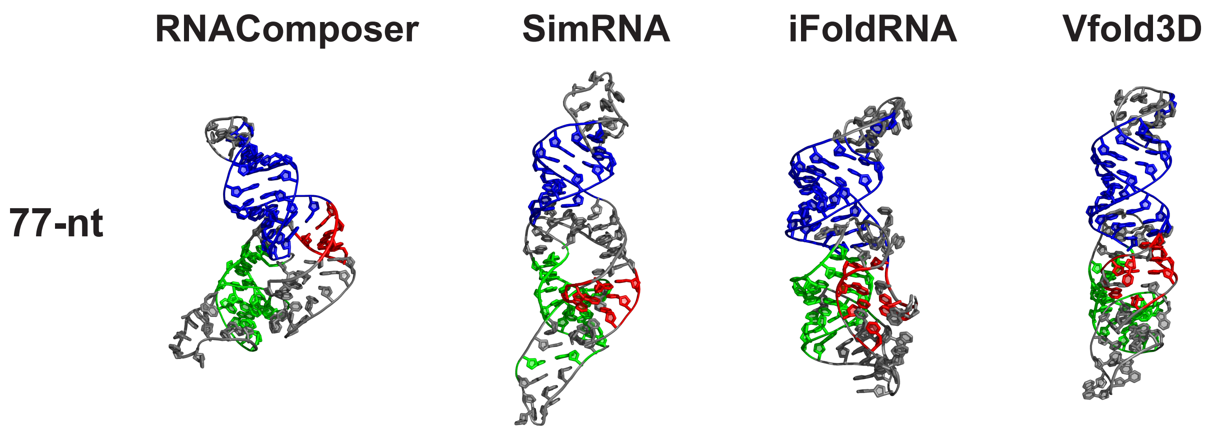

Figure S7: Largest cluster center structures of wildtype 3_5 junction for 77-nt using different 3D structure prediction programs. Stem 1 is colored blue, Stem 2 red, and Stem 3 green.

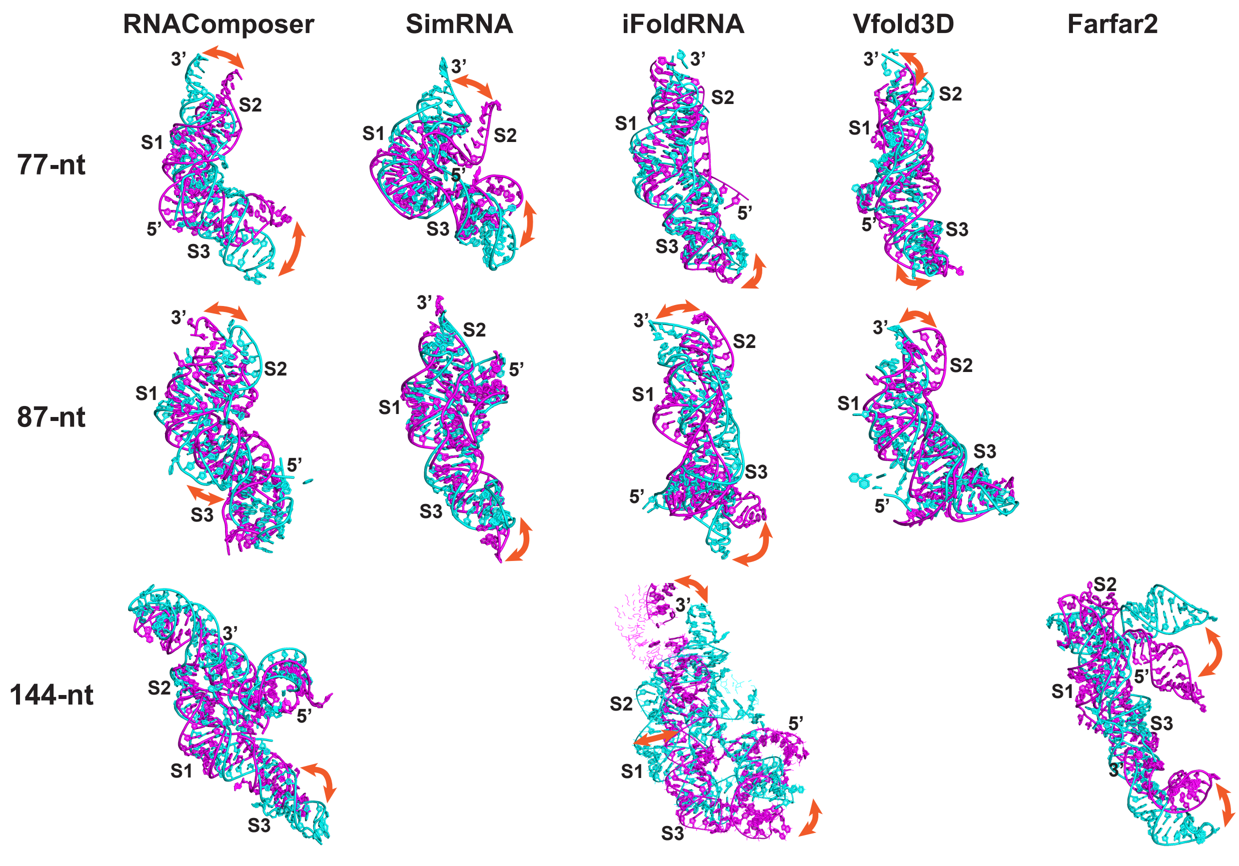

Figure S8: Dominant motions in the wildtype 3_6 pseudoknot systems revealed by principal component analysis. Two extreme frames are extracted and colored in magenta and cyan. The stems and the 5′ and 3′ ends are labeled, and the motions are highlighted using arrows. In the 144-nt iFoldRNA structure, some of residue distances in the two extreme frames are higher than usual, and these residues can only be visualized using line drawing method in PyMol.

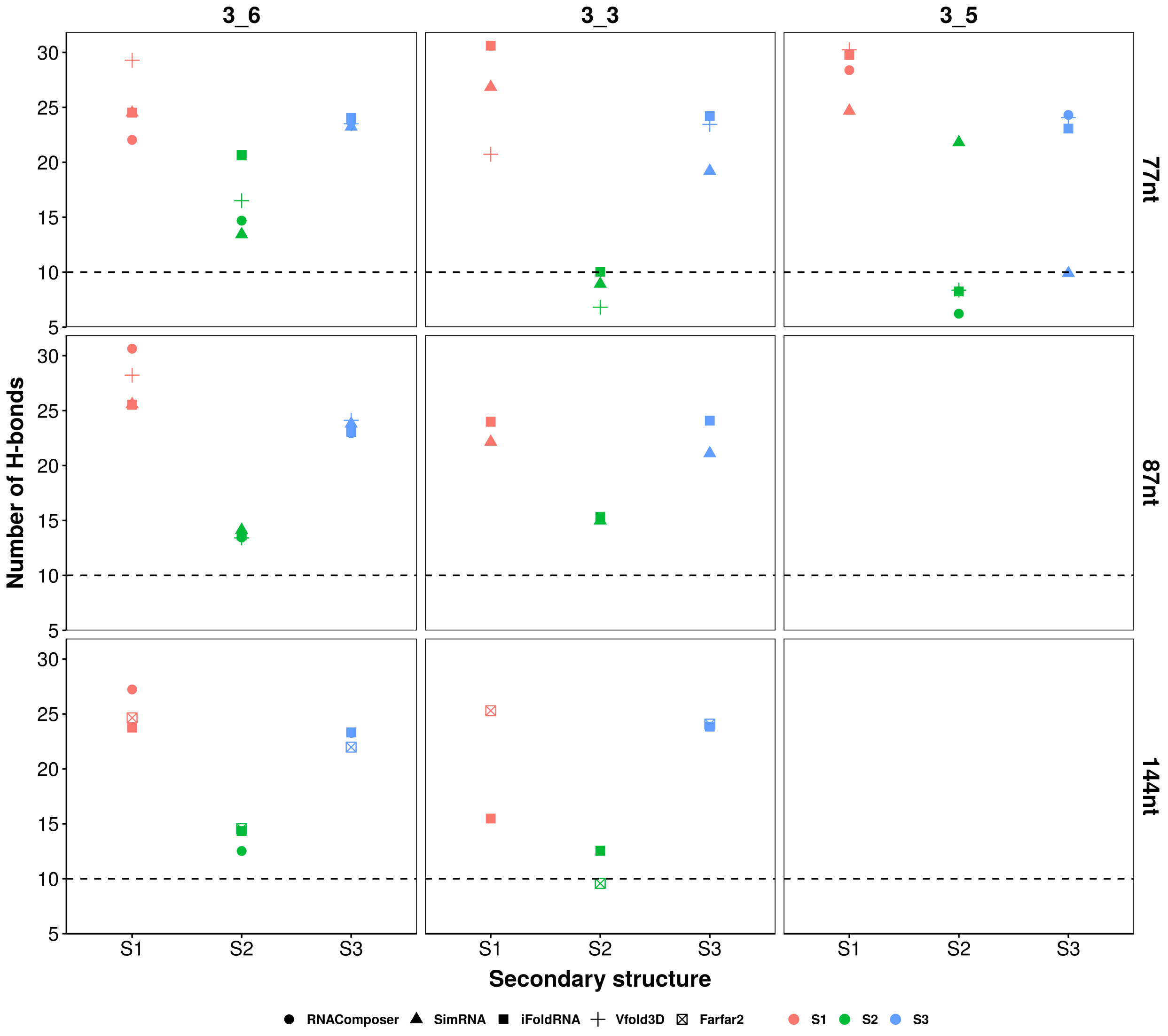

Figure S9: Number of H-bonds in the three stems of the wildtype 3_6, 3_3, and 3_5 conformations for 77, 87, and 144-nt. Stem 2 is weakest in all three conformations. At 77-nt, 3_6 has the strongest Stem 2. At 87-nt, 3_3 has a slightly stronger Stem 2 than 3_6.

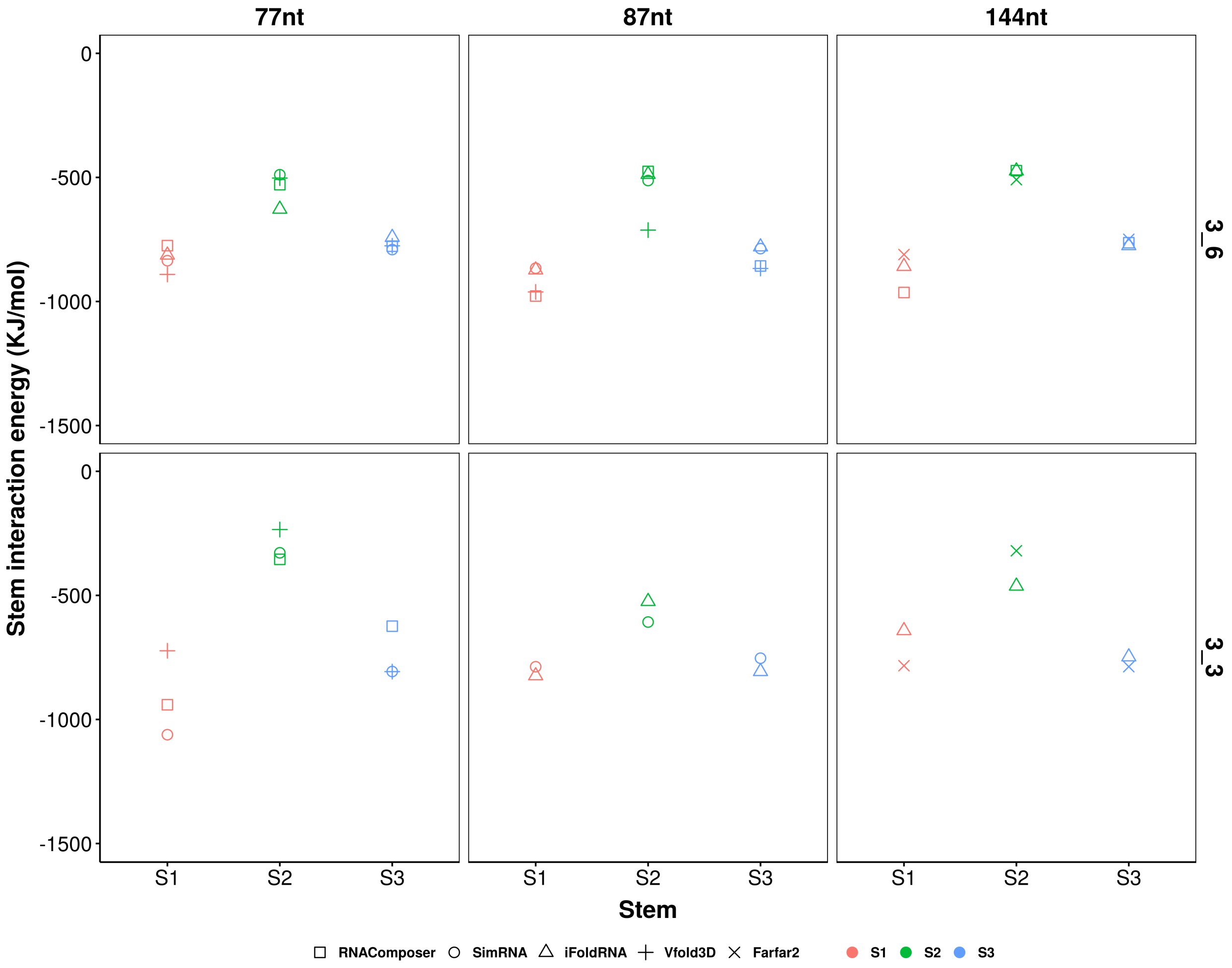

Figure S10: Interaction energy between the strands in the three stems of the wildtype 3_6 and 3_3 conformations for 77, 87, and 144-nt.

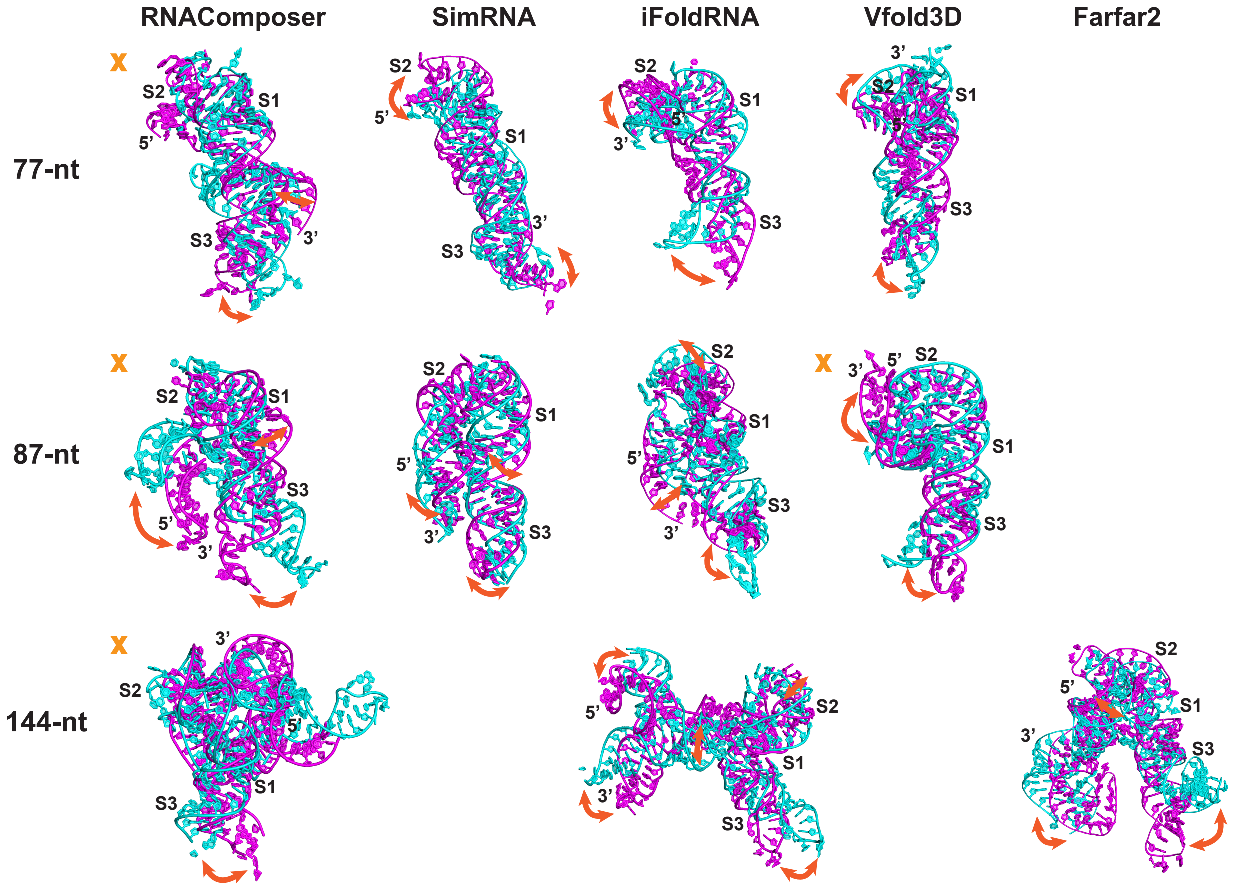

Figure S11: Dominant motions in the wildtype 3_3 pseudoknot systems revealed by principal component analysis. Two extreme frames are extracted and colored in magenta and cyan. The stems and the 5′ and 3′ ends are labeled, and the motions are highlighted using arrows. The 77-nt RNAComposer, the 87-nt RNAComposer and Vfold3D, and the 144-nt RNAComposer systems fail to form 3_3 Stem 2, and they are marked by an “x”.

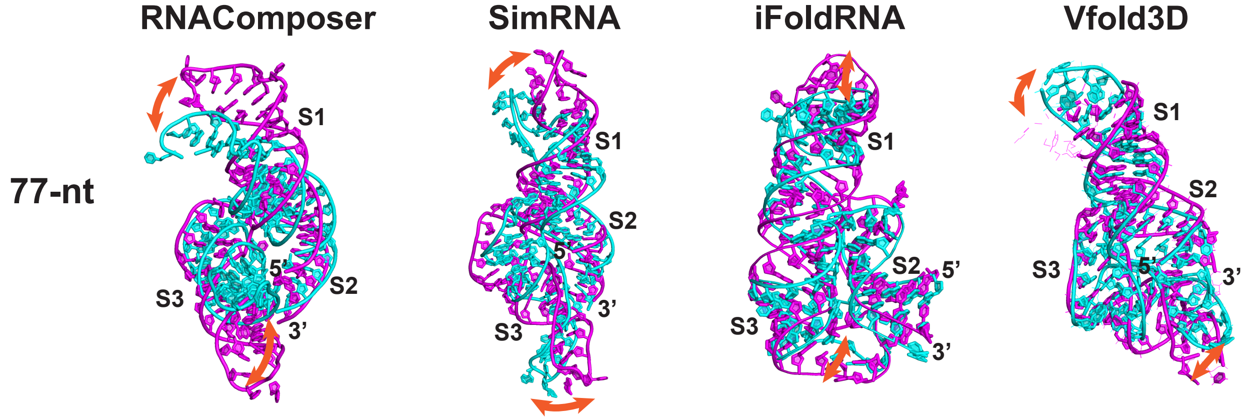

Figure S12: Dominant motions in the wildtype 3_5 junction systems revealed by principal component analysis. Two extreme frames are extracted and colored in magenta and cyan. The stems and the 5′ and 3′ ends are labeled, and the motions are highlighted using arrows. In the Vfold3D structure, some of residue distances in the two extreme frames are higher than usual, and these residues can only be visualized using line drawing method in PyMol.

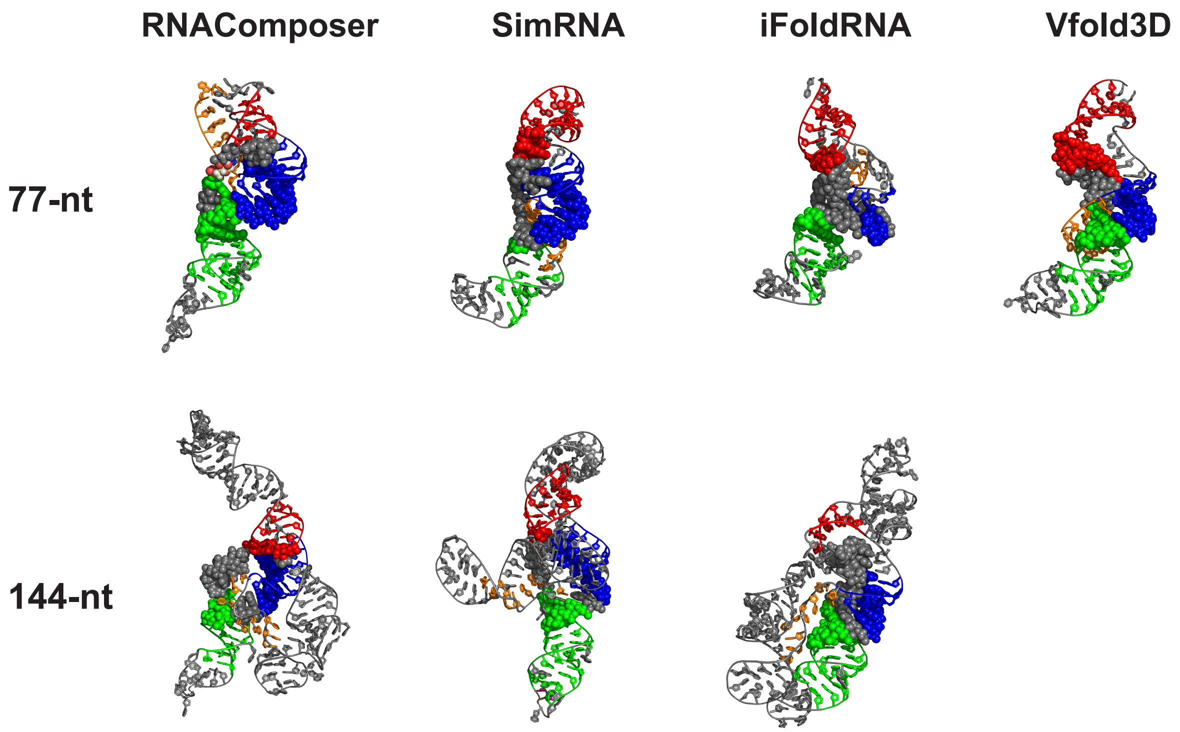

Figure S13: Largest cluster center structures of mutant 3_6 pseudoknot for lengths 77 and 144-nt using different 3D structure prediction programs. Stem 1 is colored blue, Stem 2 red, and Stem 3 green. The ring formed by the 3′ strand of Stem 1, the Stem 2/3 junction, and the Stem 1/3 junction is highlighted. The 5′ strand of Stem 1 and the 5′ end are colored orange, and in some systems, they thread through the ring.

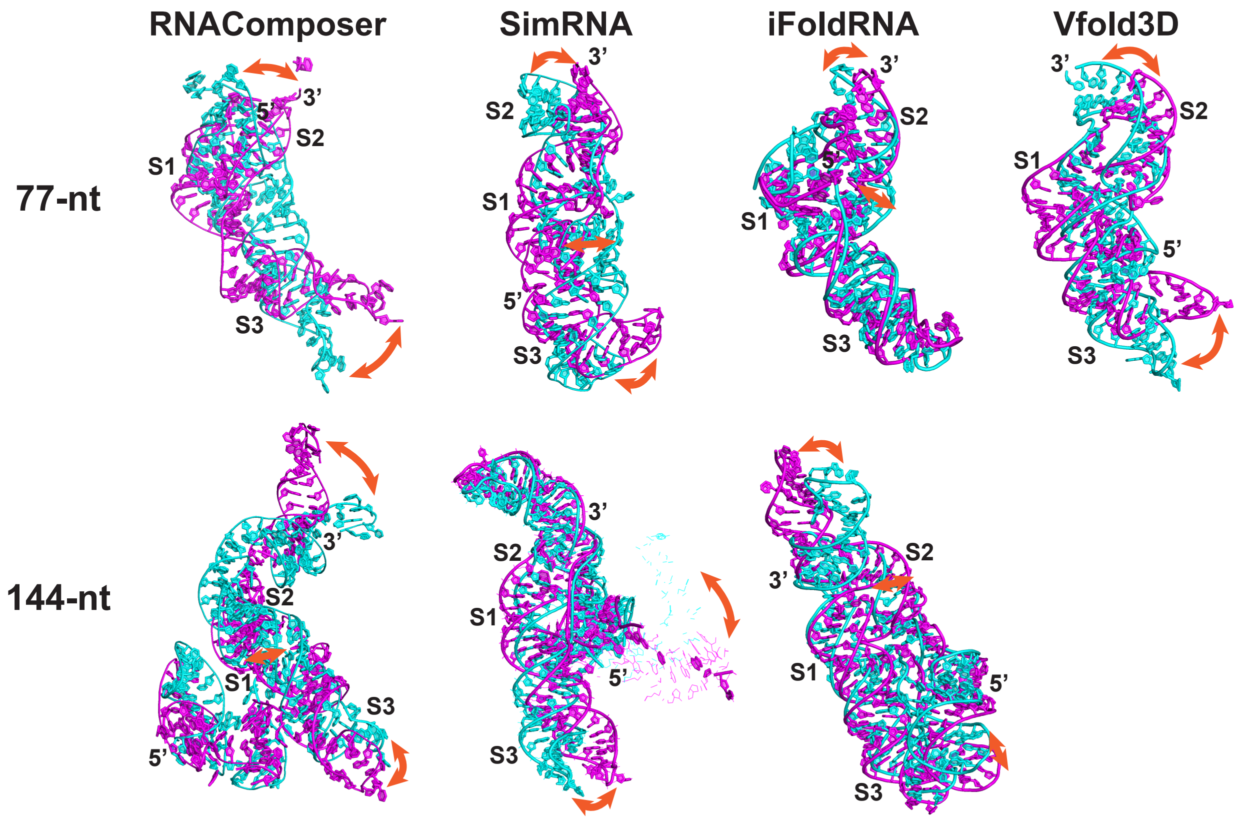

Figure S14: Dominant motions in the mutant 3_6 pseudoknot systems revealed by principal component analysis. Two extreme frames are extracted and colored in magenta and cyan. The stems and the 5′ and 3′ ends are labeled, and the motions are highlighted using arrows. In the 144-nt SimRNA structure, some of residue distances in the two extreme frames are higher than usual, and these residues can only be visualized using line drawing method in PyMol.

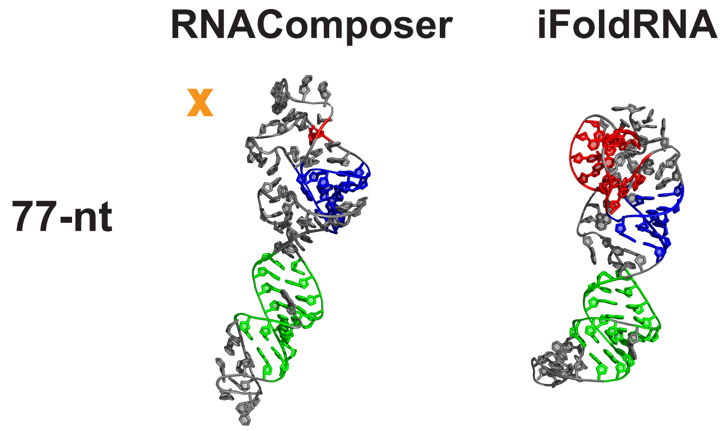

Figure S15: Largest cluster center structures of mutant 3_3 pseudoknot for lengths 77-nt using different 3D structure prediction programs. Stem 1 is colored blue, Stem 2 red, and Stem 3 green. The RNAComposer system fails to form 3_3 Stem 2, and is marked by an “x”.

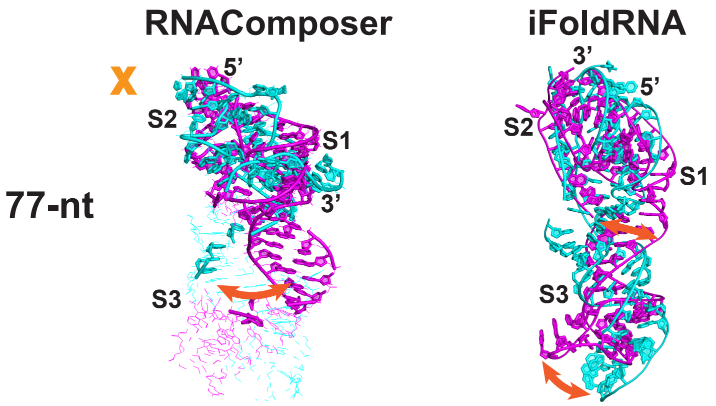

Figure S16: Dominant motions in the mutant 3_3 pseudoknot systems revealed by principal component analysis. Two extreme frames are extracted and colored in magenta and cyan. The stems and the 5′ and 3′ ends are labeled, and the motions are highlighted using arrows. In the RNAComposer structure, some of residue distances in the two extreme frames are higher than usual, and these residues can only be visualized using line drawing method in PyMol. The RNAComposer system fails to form 3_3 Stem 2, and is marked by an “x”.

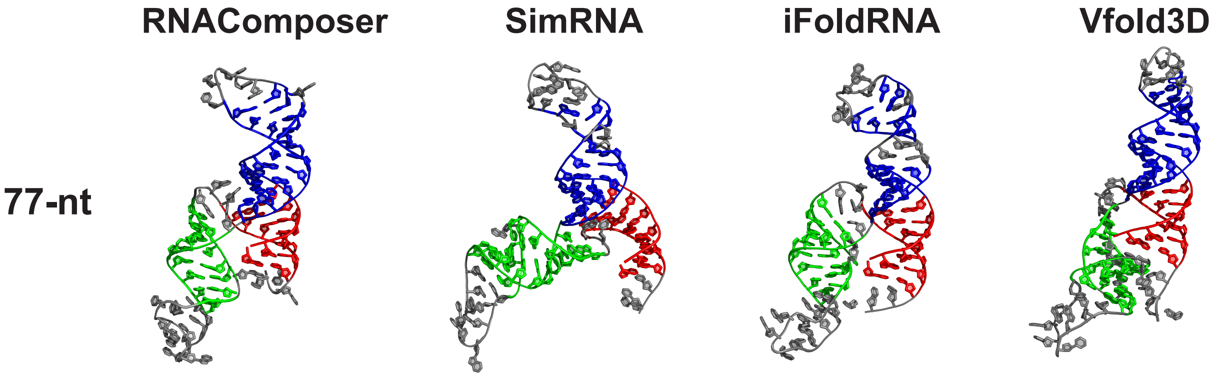

Figure S17: Largest cluster center structures of mutant 3_5 junction for 77-nt using different 3D structure prediction programs. Stem 1 is colored blue, Stem 2 red, and Stem 3 green.

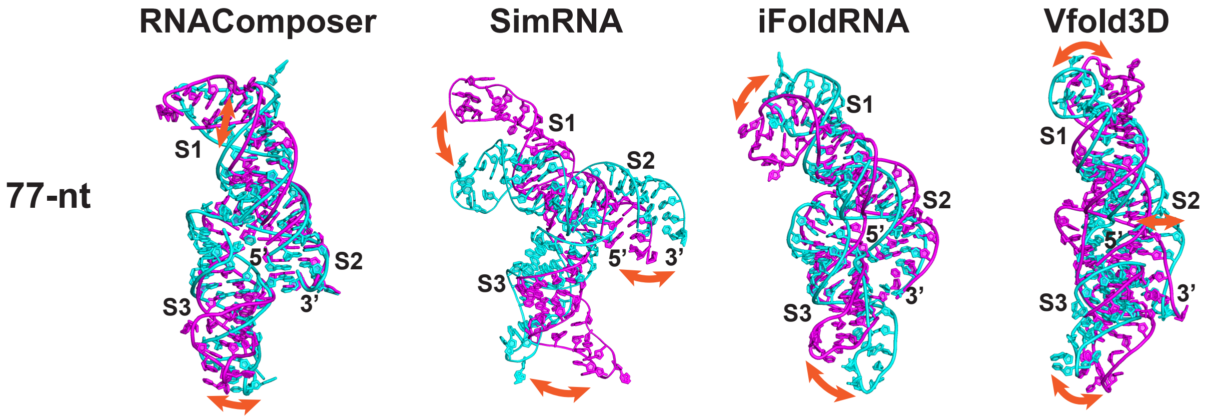

Figure S18: Dominant motions in the mutant 3_5 junction systems revealed by principal component analysis. Two extreme frames are extracted and colored in magenta and cyan. The stems and the 5′ and 3′ ends are labeled, and the motions are highlighted using arrows.

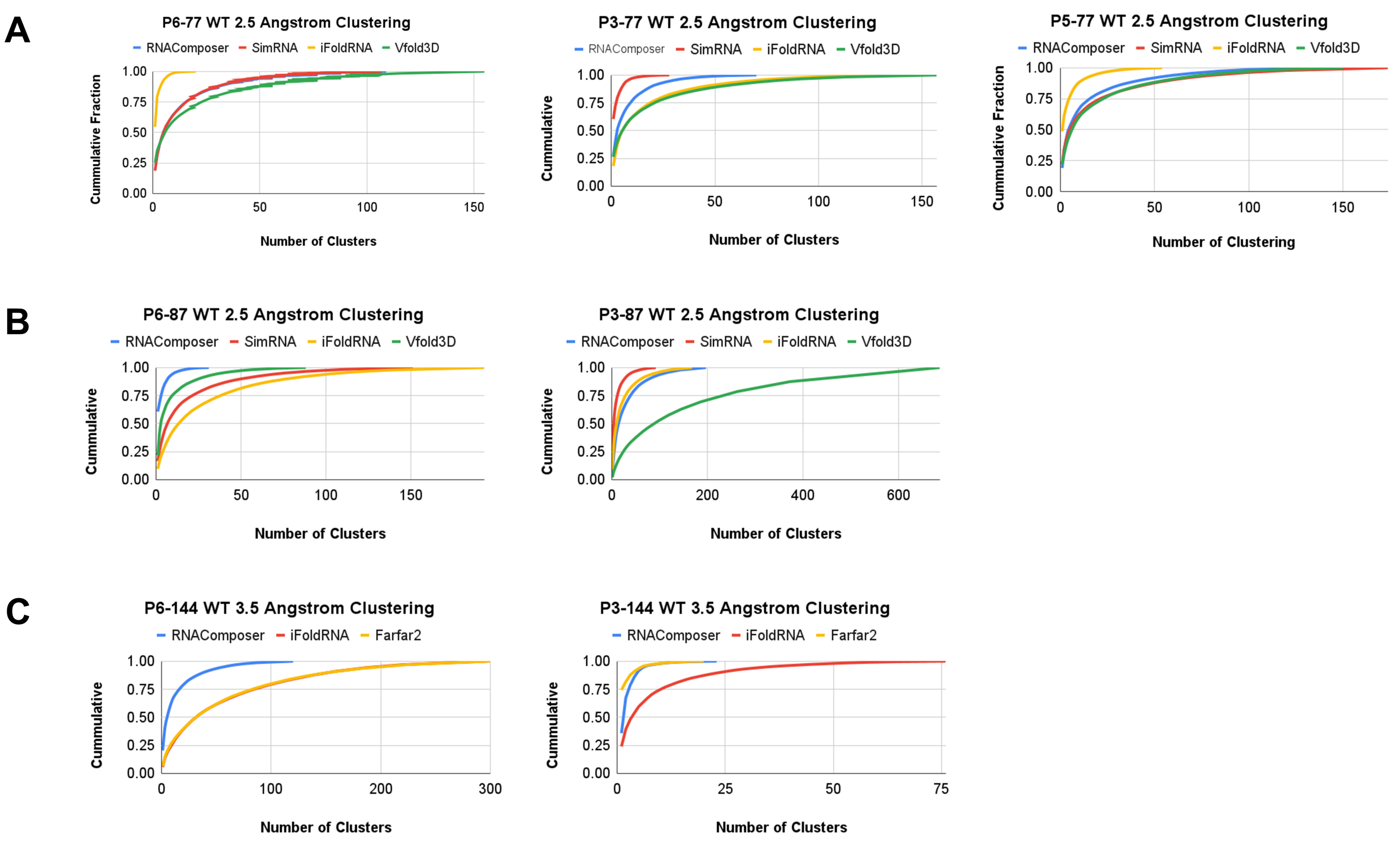

Figure S19: Cluster analysis of the FSE MD simulations for (A) 77-nt, (B) 87-nt, and (C) 144-nt systems modeled in this work. The systems are named based on the FSE conformation (*P*6 for 3_6, *P*3 for 3_3 and *P*5 for 3_5). Cumulative fraction of the structures is calculated as the number of clusters increases. Cluster size is calculated for each system to compare the structural variation among copies of simulations as well as to track convergence of MD. The clusters are ranked from the largest to the smallest in size. To make sure that a feasible number of clusters exist in all the systems of the same length, cutoff is defined to be 2.5 Å for 77 and 87-nt, or 3.5 Å for 144-nt.

References

(1) Elbe, S.; Buckland-Merrett, G. Data, disease and diplomacy: GISAID's innovative contribution to global health. *Glob. Chall.* **2017**, *1*, 33–46.

(2) Schlick, T.; Zhu, Q.; Dey, A.; Jain, S.; Yan, S.; Laederach, A. To Knot or Not to Knot: Multiple Conformations of the SARS-CoV-2 Frameshifting RNA Element. *J. Amer. Chem. Soc.* **2021**, *143*, 11404–11422.
